# Suitability mapping of native tree species in dry-hot valleys of Yunnan based on InVEST-MaxEnt coupled modeling: model validation framework with native tree species actual distribution and seed germination

**DOI:** 10.3389/fpls.2025.1577623

**Published:** 2025-04-25

**Authors:** Meng Xie, Xiaobo Song, Xuexing Zhang, Yongpeng Ma, Zhilin Song, Fengjuan Li, Wei Li, Linyuan Fan, Hong Ma

**Affiliations:** ^1^ Institute of Highland Forest Science, Chinese Academy of Forestry, State Key Laboratory of Efficient Production of Forest Resources, Yunnan, Kunming, China; ^2^ Rushan Inspection and Testing Center, Rushan, China; ^3^ Yunnan Academy of Forestry and Grassland, Kunming, China; ^4^ Key Laboratory of Comprehensive Conservation for Extremely Small Populations of Wild Plants in Yunnan Province, Kunming Institute of Botany, Chinese Academy of Sciences, Kunming, China; ^5^ Rushan Forestry Development Center, Rushan, China; ^6^ Yunnan Jicheng Landscape Technology Co., Ltd., Mile, China; ^7^ Yunnan General Administration of Forestry Seeds and Seedlings, Kunming, China; ^8^ Key Laboratory of Breeding and Utilization of Resource Insects, National Forestry and Grassland Administration, Kunming, China; ^9^ Yuanmou Desert Ecosystem Research Station, National Long-Term Scientific Research Base of Comprehensive Control, Chuxiong, Yunnan, China

**Keywords:** dry-hot valleys, native tree species, InVEST-MaxEnt coupled modeling, suitability mapping, model validation framework

## Abstract

**Introduction:**

The target valleys along the Jinsha, Nujiang, Lancang, and Yuanjiang Rivers exhibit acute human-land conflicts and ecosystem vulnerability. Predicting the distribution of potential suitable habitats for native tree species in Yunnan Province provides basin-scale insights for the management of ecosystems in dry and hot valleys, thereby advancing restoration planning in dry-hot valleys.

**Methods:**

This study investigates native tree species suitability in Yunnan’s dry-hot valleys using an integrated MaxEnt-InVEST modeling framework.

**Results:**

Temperature and precipitation emerged as dominant bioclimatic controls, with optimal species occurrence (1 000–2 500 m) showing negative elevation correlation. Four native tree species (*Osteomeles schwerinae*, *Phyllanthus emblica*, *Quercus francetii* and *Sapindus delavayi*) displayed fragmented suitable areas along mountainous riparian zones, while habitat quality hotspots mainly covered non-urbanized regions, avoiding central urban clusters and northeastern/southeastern karst zones. The coupled model demonstrated significantly improved accuracy compared to the standalone MaxEnt by incorporating land-use impacts, with Yuanmou County case analysis confirming the enhanced predictive capability through actual distribution patterns. Spatial prioritization identified core planting clusters in central/southeastern valleys, though fragmented by agricultural encroachment.

**Discussion:**

This methodology provides a cost-effective solution for vegetation restoration planning in ecologically fragile dry-hot ecosystems. The research results can provide scientific support for the restoration of degraded ecosystems in dry-hot valleys of Yunnan Province, the national Afforestation program and soil and water conservation projects.

## 1 Introduction

Plant distribution is primarily governed by abiotic factors, while climate change is accelerating the restructuring of distribution patterns (Soberón, 2007). Global climate change has exacerbated extreme drought events, particularly in Yunnan Province, where escalating drought frequency and intensity pose severe constraints on vegetation restoration (Jia and Pan, 2016; Cook et al., 2020). The uniqueness of dry-hot valleys lies in their extreme habitat: annual evaporation can exceed precipitation by 3 to 6 fold, coupled with nutrient-depleted soils and seasonal high temperatures (>40°C), forming an “ecological bottleneck” for vegetation rehabilitation (Liu and Yin, 2013). Despite hosting nearly 2,000 plant species (including 46 endemic taxa) in Yunnan’s dry-hot valleys (Jin, 1999), climate change may reduce suitable habitats for 70% of species by over 30%, highlighting the urgency of identifying drought-tolerant species (Zhang et al., 2014; Zhao and Gong, 2015).

Understanding plant responses to climate change constitutes the scientific foundation for formulating regional conservation strategies, critical for maintaining biodiversity in dry-hot valleys and enhancing the long-term sustainability of forest restoration policies. Evaluating the growth capacity and habitat suitability of tree species in ecologically fragile areas provides essential insights for guiding ecological restoration efforts (Blakesley et al., 2002; Elliott et al., 2003). Climate-based species distribution models (SDMs), which mathematically correlate species niches with environmental variables, offer robust predictions of potential suitable habitats under climate change scenarios (Dhyani et al., 2023; Li et al., 2023). Among these models, the maximum entropy (MaxEnt) model has emerged as the most widely adopted tool due to its superior predictive accuracy and stability (Phillips et al., 2006; Boral and Moktan, 2021). According to MaxEnt modeling principles, candidate tree species demonstrating maintained or increased habitat suitability probabilities in dry-hot valleys under current and projected climate conditions are likely to possess climate adaptation potential. Notably, MaxEnt predictions may be influenced by localized microclimates and anthropogenic disturbances (e.g., land-use policies), while remaining unable to assess species’ reproductive success—a critical limitation given that seed germination and seedling establishment are more environmentally sensitive than adult survival (Grubb, 1977; McLaughlin and Zavaleta, 2012). Therefore, validating predicted suitability against species’ early-life-stage requirements and natural distribution patterns of native tree species is imperative. Field surveys in target restoration areas are further recommended to reconcile discrepancies between predicted habitat suitability and observed survival outcomes.

Current studies on habitat suitability predictions for plants in Yunnan’s dry-hot valleys predominantly focus on single tree species (Ying et al., 2016), with existing research largely limited to biodiversity assessments (Liu et al., 2020), species introduction evaluations (Ma and McConchie, 2001), and vegetation restoration applications (Yang et al., 2016). Few investigations have systematically integrated habitat suitability modeling into species selection protocols for ecological restoration. The Habitat Quality module within the InVEST model employs a multi-threat analysis framework to quantify ecosystem resilience. Utilizing land use/cover data, it systematically characterizes habitat typologies and anthropogenic/natural stressors in dry-hot valleys. The algorithm calculates habitat degradation through spatialized threat intensity assessment, subsequently integrating habitat suitability indices to derive habitat quality metrics. This integrative assessment framework evaluates both biophysical resource accessibility and ecosystem stability maintenance capacity, serving as a critical proxy for biodiversity conservation potential (Xu et al., 2019). The synergistic analysis of land-use-derived proxy indicators (e.g., habitat quality) and MaxEnt-based habitat suitability predictions represents a cost-effective conservation strategy, balancing ecological protection and management efficiency (Gong et al., 2019; Watson et al., 2020). This approach holds particular relevance for the dry-hot valley ecosystem, where severe degradation and intensified habitat fragmentation have significantly compromised biodiversity and regional sustainable development (Yang et al., 2016; Zhu et al., 2020). By modeling interactions between climate change, urbanization pressures, and extant biodiversity, this framework provides critical insights for optimizing conservation efforts in ecologically vulnerable regions subject to intense anthropogenic disturbances.

To address these challenges, this study establishes a novel framework integrating the Maximum Entropy (MaxEnt) model with the Integrated Valuation of Ecosystem Services and Tradeoffs (InVEST) model. Our objectives are threefold: to generate spatially explicit suitability maps for native tree species in dry-hot valleys through coupled habitat suitability and habitat quality analyses; to systematically identify conservation priority areas while evaluating the spatial congruence between multi-species suitability patterns, actual species distributions, and seed germination dynamics; and to validate the synergistic application of MaxEnt-InVEST integration for precision screening of restoration species. *O. schwerinae*, *P. emblica*, *Q. franchetii*, and *S. delavayi* are all drought-tolerant pioneer plants that can be used to restore ecologically sensitive areas in the dry-hot valleys. The four species cover the vertical levels of “shrubs-subtrees-trees” to form a coordinated drought-resistant system. The methodology combines optimized MaxEnt modeling with multi-source geospatial analysis. Species distribution models were calibrated using preprocessed occurrence records and environmental variables, subsequently overlain with current land use patterns (particularly soil-water conservation stable areas) through GIS spatial superposition. Parallel habitat quality assessments were conducted using InVEST’s habitat degradation module to quantify ecosystem vulnerability. Field validation encompassed two components: ground-truthing of model predictions through systematic plot surveys at designated restoration sites, and comparative germination trials under current versus projected aridification scenarios. This dual-scale verification strategy – contrasting modeled suitability against both extant vegetation patterns and experimental germination responses-enables robust evaluation of model predictive capacity. The resultant framework advances dry-hot valley restoration planning by enabling climate-resilient species selection and providing basin-scale insights for ecosystem management. This study can provide data support for ecological red line protection and biodiversity restoration projects in dry-hot valleys of Yunnan, and help optimize afforestation tree species selection strategies.

## 2 Materials and methods

### 2.1 Study area

**Figure 1:**
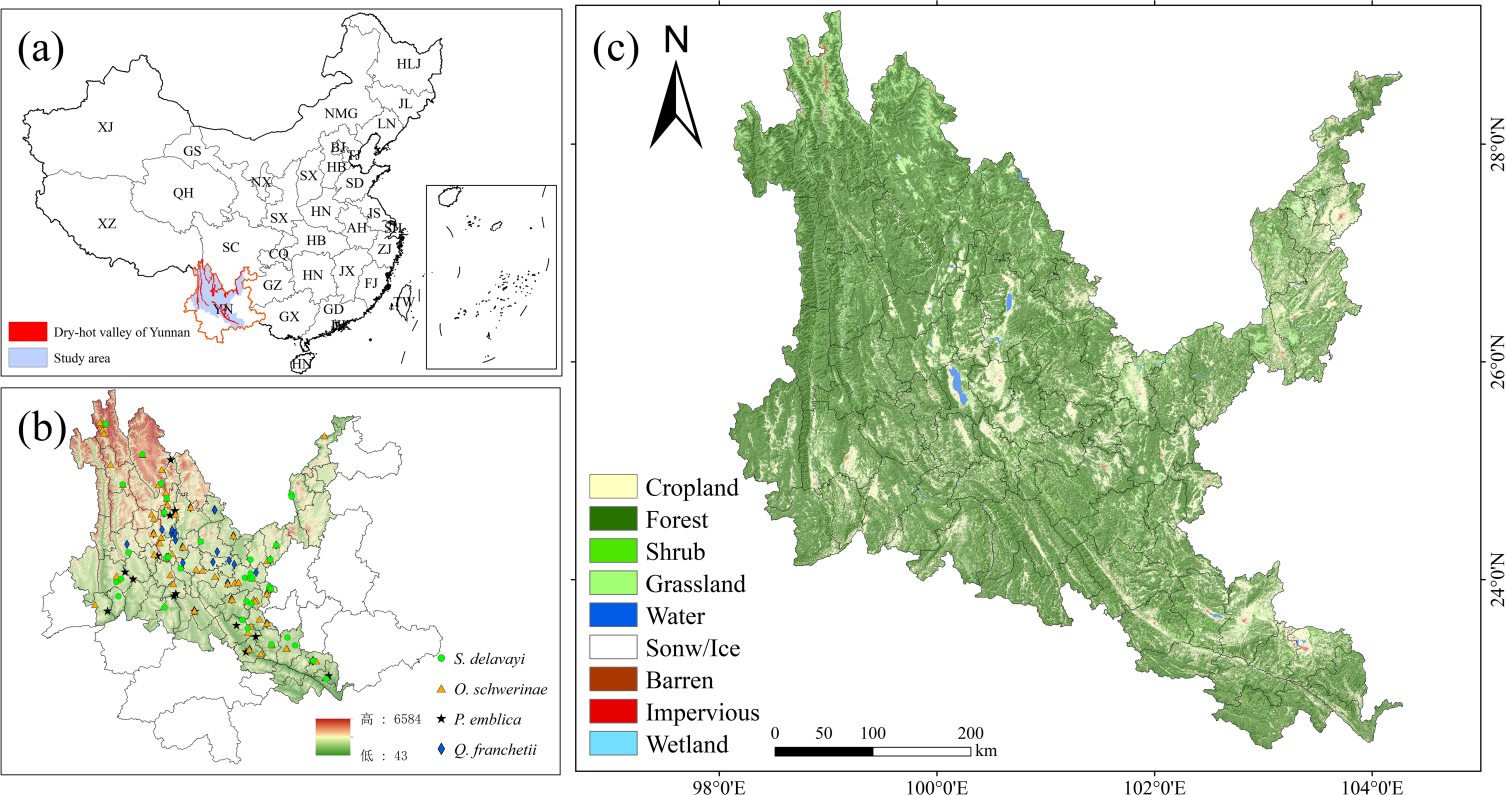
Study area (dry-hot valleys). **(a)** location of Yunnan. **(b)** location of the dry-hot valleys. **(c)** land use/land cover map of the dry-hot valleys in 2022. The Chinese Provinces are abbreviated as follows: YN, Yunnan; GX, Guangxi; GD, Guangdong; HN^a^, Hainan; TW, Taiwan; FJ, Fujian; ZJ, Zhejiang; JX, Jiangxi; JS, Jiangsu; AH, Anhui; HN^b^, Hunan; HB^a^, Hubei; HN, Henan; HB, Hebei; SX^a^, Shanxi; D, Shandong; GZ, Guizhou; CQ, Chongqing; SC, Sichuan; XZ, Xizang; XJ, Xinjiang; QH, Qinghai; GS, Gansu; NX, Ningxia; SX, Shaanxi; NMG, Neimenggu; LN, Liaoning; JL, Jilin; HLJ, Heilongjiang.

The dry-hot valleys of Yunnan Province, China (23°00′-28°10′N, 98°50′-103°50′E), encompass approximately 13,800 km²of mountainous terrain along the Jinsha, Nujiang, Lancang, and Yuanjiang river systems (Fan et al., 2020). These valleys exhibit discontinuous spatial configurations at elevations ranging from 500 to 1,600 m (**Figure 1**), characterized by a tropical monsoon-influenced arid climate with pronounced seasonal aridity, extreme diurnal temperature fluctuations, and steep altitudinal gradients. The region supports semi-savanna vegetation dominated by xerophytic species demonstrating exceptional ecological adaptation, notably *P. emblica*, *Dodonaea viscosa* and *Heteropogon contortus* (Yang et al., 2016; Fan et al., 2020). This unique ecotone represents one of southwestern China’s most climate-sensitive and ecologically vulnerable landscapes, where vegetation restoration faces dual challenges of hydrothermal stress and soil degradation.

### 2.2 Research framework

**Figure 2:**
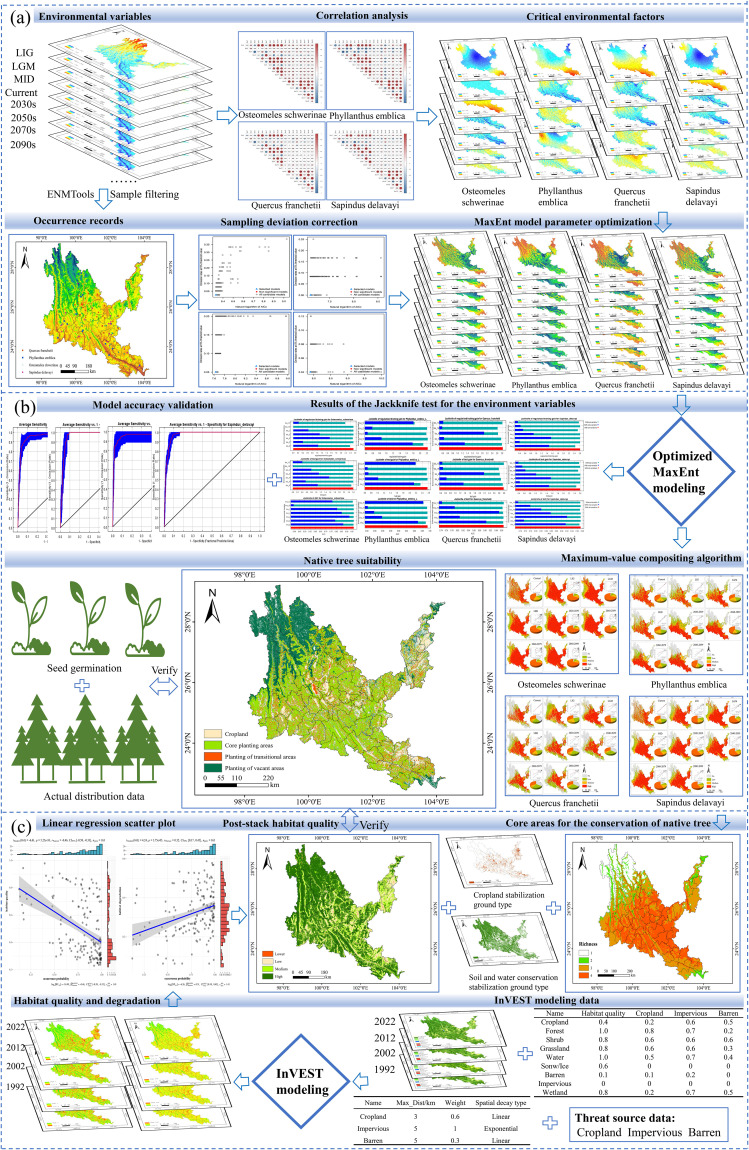
Research framework. **(a)** data collection, preprocessing, and MaxEnt model optimization. **(b)** Prediction mapping of the suitability of native tree species. **(c)** InVSET model data collection and model accuracy validation. LIG, last inter-glacial; LGM, last glacial maximum; MID, mid holocene.

The research framework consists of three steps (**Figure 2**): (a) Obtaining data on the occurrence sites of native tree species and environmental variables. Using the ENMTools tool to remove data that does not conform to the range of environmental variables, analyze the correlation between environmental variables, and reduce data redundancy. (b) Optimizing sampling bias and model parameters, and verifying the accuracy of the model. (c) The preprocessed data and the optimized MaxEnt model were used to simulate the planting suitability of different native tree species, superimposed on the current land use status (soil and water conservation stable land type), and combined with the InVEST model to analyze the habitat quality and degradation degree of dry-hot valleys and evaluate their consistency.

### 2.3 MaxEnt prediction

#### 2.3.1 Record of occurrence sites of native tree species

**Figure 3:**
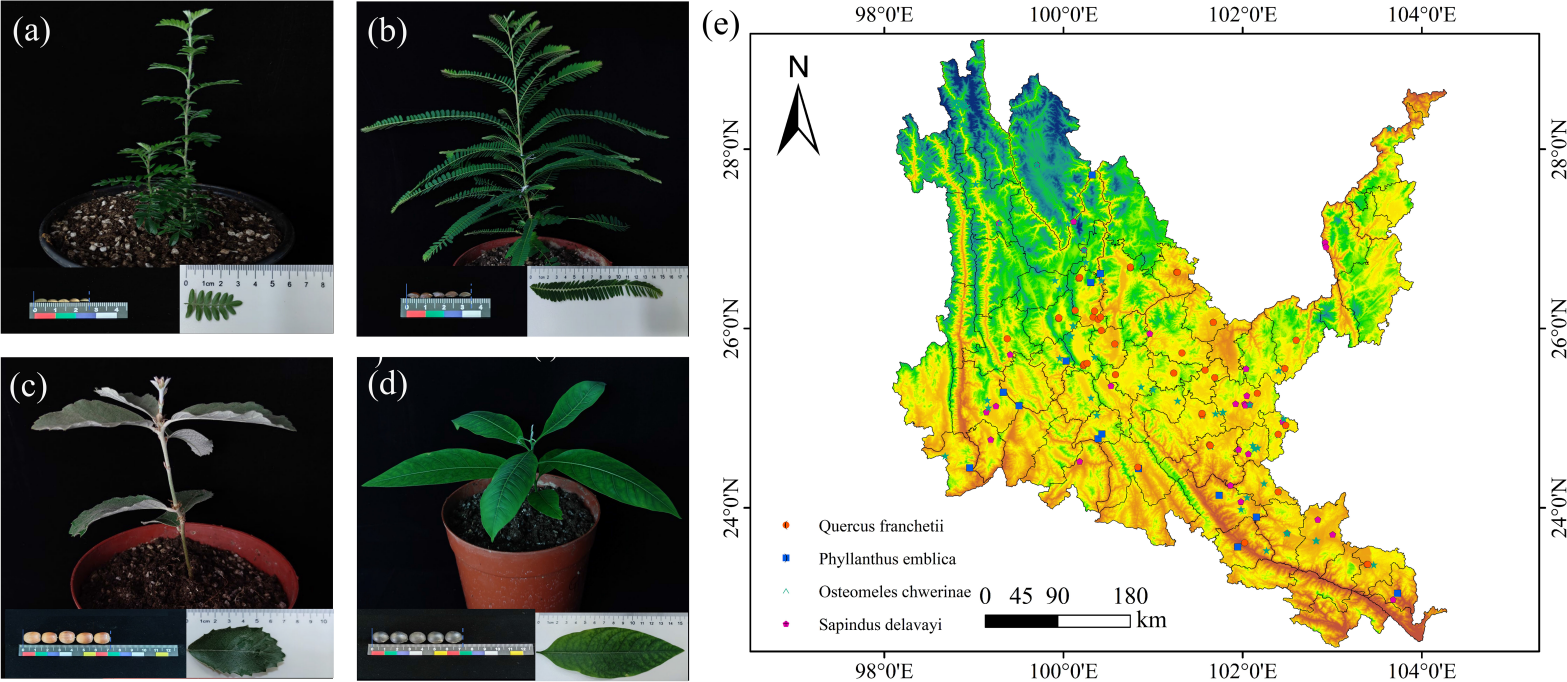
Seed, leaf, and seedling photographs and their geographical distribution of native tree sprcies in dry-hot valleys of Yunnan. **(a)** *Osteomeles schwerinae*. **(b)** *Phyllanthus emblica*. **(c)** *Quercus franchetii*. **(d)** *Sapindus delavayi*. **(e)** Red dots indicate occurrence records of *Quercus franchetii*; blue dots, *Phyllanthus emblica*; green dots, *Osteomeles schwerinae*; pink dots, *Sapindus delavayi*.

The tree species involved in the study mainly refer to the four native tree species selected by the research team based on field investigations, combined with information such as the *Flora of Yunnan* (Yunnan Institute of Botany, 1977), *Study on the selection of indigenous plants in Southwest China* (Ma et al., 2019), and *the List of Major Native Tree Species in Yunnan Province (First Batch)* (http://lcj.yn.gov.cn/html/2021/gongshigong gao_0916/63894). The distribution data come from the Global Biodiversity Information Facility (http://www.gbif.org (accessed on March 20, 2023)), the China National Specimen Information Infrastructure (http://www.naii.org.cn (accessed on March 21, 2023)), the Chinese Virtual Herbarium (http://www.cvh.ac.cn (accessed on March 21, 2023)) and the field survey data of the research team. The distribution data years are 1970-2023. To avoid overlapping of distribution data and remove erroneous duplicates and data that are not within the dry hot valley domain, the ENMTools tool was used to filter so that only one data was retained in each grid (1 km × 1 km) (**Figure 3**).

#### 2.3.2 Environmental factor selection and pretreatment

Based on 19 environmental factors, the study predicted its distribution pattern in eight periods, namely the Last inter-glacial (LIG), Last Glacial Maximum (LGM), Mid Holocene (MID), Current and future (2030s, 2050s, 2070s and 2090s). The 19 climate data were sourced from the World Climate Data (http://www.woldclim.org). The future climate data were selected from the BCC-CSM2-MR climate model in CMIP6 that is suitable for China’s geographical environment, and the moderate development path (SSP245) that limits warming to less than 3°C was selected (Zhang et al., 2023). The paleoclimate data of LIG, LGM, and MID were downloaded from the WorldClim 1.4 dataset. The administrative map of China and the base map of Yunnan Province were obtained from the website of the National Catalogue Service for Geographic Information (www.webmap.cn). The correlation between environmental factors directly leads to overfitting of the MaxEnt model results, affecting the accuracy of the contribution rate of environmental factors and the potential distribution area of species. To mitigate multicollinearity risks in the MaxEnt model, an environmental variable optimization strategy was implemented: Initial modeling was conducted using 19 bioclimatic variables, with non-sensitive variables (zero contribution rate) eliminated through Jackknife test. Pearson correlation analysis (|r| ≥0.8 defined as high correlation) was then applied to retained variables to establish variable selection criteria, prioritizing retention of variables with higher contribution rates and clear ecological relevance among highly correlated pairs (De Marco and Nóbrega, 2018). After completing the above comparison steps, the corresponding variables of each native tree species were retained as environmental factors for predicting its distribution (**Supplementary Table S1**).

### 2.4 MaxEnt model optimization

Parameter optimization for the MaxEnt model was implemented in R 3.6.3 using the “kuenm” package (Cobos et al., 2019; available at: https://github.com/marlonecobos/kuenm). A total of 1,160 candidate models were generated by systematically exploring 29 permutations of feature combinations (FC: linear [L], quadratic [Q], product [P], threshold [T], and hinge [H]) across regularization multipliers (RM: 0.1-4.0 with 0.1 increments), following established protocols (Warren et al., 2014; Muscarella et al., 2014). Optimal parameter sets were selected using the delta AICc criterion, which balances model complexity and goodness-of-fit (Warren and Seifert, 2011; Shcheglovitova and Anderson, 2013).

### 2.5 Model simulation and accuracy verification

Species distribution modeling was conducted using MaxEnt 3.4.4 (https://biodiversityinformatics.amnh.org/open_source/maxent/), incorporating preprocessed species occurrence data and environmental variables. The model was configured with 75% training data and 25% testing data across 10 cross-validation replicates. Model performance was evaluated through Jackknife tests and ROC curve analysis, with predictive accuracy validated by AUC values (>0.6 indicating acceptable performance). Outputs were processed in ArcGIS 10.8 to classify habitat suitability into four tiers (0–0.2: unsuitable; 0.2–0.4: low suitable; 0.4–0.6: medium; 0.6–1.0: highly suitable) and analyze spatiotemporal dynamics of core suitable habitats via raster calculus and multitemporal overlay (Yang et al., 2013). Stable high-suitability zones (≥0.6) and moderate-suitability zones (0.4–0.6) derived from multitemporal probability superposition were identified as priority conservation hotspots for dry-hot valley tree species. Stable high-suitability zones (≥0.6) and moderate-suitability zones (0.4–0.6) derived from multitemporal probability superposition were identified as priority conservation hotspots for dry-hot valley tree species (Xie et al., 2024).

### 2.6 InVEST model construction

**Figure 4:**
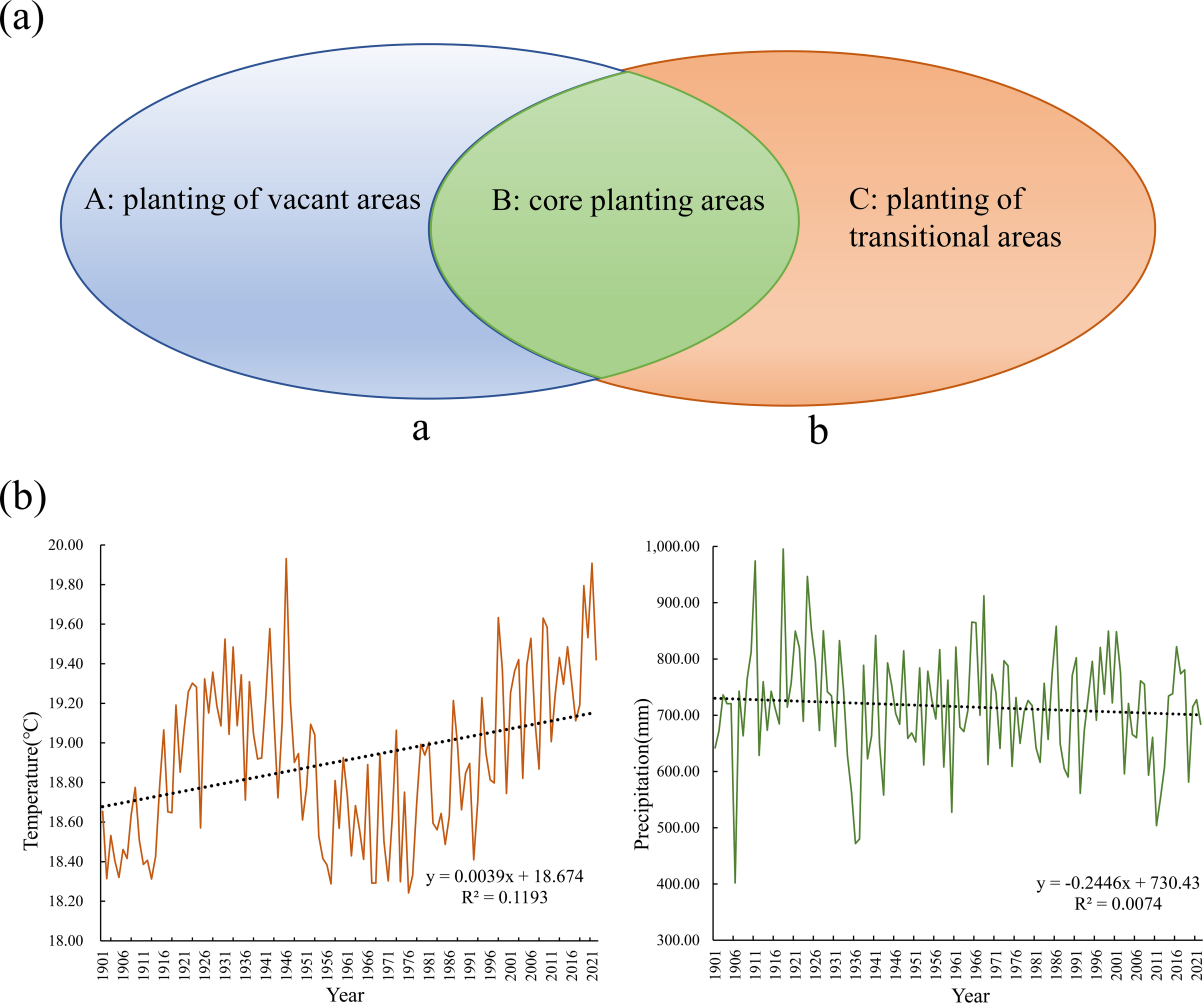
Zoning of Hotspot Areas and Variation Patterns of Temperature and Precipitation in Yuanmou County. **(a)** Regional division. a: soil and water conservation stabilization areas, b: core area of native tree species in the dry-hot valleys. (A): located in **(a)** but not in B; (B): located in the area of overlap between **(a, b)**; (C): located in **(b)** but not in B and not part of the cropland stabilization area. **(b)** changes in mean annual climate normals for temperature and precipitation in Yuanmou County over the last 100a.

This research identified cultivated lands, built-up areas, and barren lands as core threat factors. A threat weighting-spatial decay matrix (**Supplementary Table S2**) and habitat sensitivity parameter system (**Supplementary Table S3**) were rigorously developed following InVEST technical specifications, with model calibration achieved through iterative K-value optimization (initial K=0.5, secondary optimization at K=0.25). Based on a four-tier habitat quality classification system (high: >0.6; medium: 0.4-0.6; low: 0.2-0.4; relatively low: <0.2), areas with moderate-high quality (≥0.4) were designated as the core areas of native tree species in the dry-hot valleys. Synergistic spatial coupling analysis of MaxEnt-InVEST models extracted overlapping hotspot regions as core native tree species habitats. Multitemporal land cover data superimposition (2000-2022) identified soil and water conservation stable land types (forest, shrub, and grassland) and farmland stable land types. A three-tier functional planting zoning framework was established: Core planting areas (complete spatial overlap between habitat hotspots and stable land types), Planting transition areas (partial ecological suitability overlap), and Planting gap areas (no ecological suitability overlap) (**Figure 4a**).

### 2.7 Field survey and seed germination analysis

Yuanmou County, Yunnan Province (2,025.58 km², population 195 200) has a subtropical hot and dry climate. The Jinsha River system contains 62 rivers (19 regular rivers/43 seasonal rivers). Dry-hot valleys account for 39.4% (797.51 km²) of the county, with an extreme temperature range of 41-1.8°C, an average annual temperature of 22.6°C, and an annual precipitation of 445.6 mm. There are 1,297 vascular plant species, 15.7% of cultivated land, and 53.1% of forest land. The century-long climate data shows a warming trend (+0.3°C/10a) and precipitation attenuation (-6.8mm/10a), which exacerbates drought (Peng, 2019; Peng, 2022) (**Figure 4b**). This study verified the accuracy of the InVEST-MaxEnt model by conducting field surveys in dry-hot valleys section of Yuanmou County, Chuxiong Prefecture, Yunnan Province, a core protected area, from 2022 to 2023, and confirmed the actual distribution of the species of *O. schwerinae*, *P. emblica*, *Q. franchetii*, and *S. delavayi* by combining daily data, opinion collection, and forest resource monitoring data from the Yuanmou County Forestry Bureau. The mature seeds were collected and processed in a standardized manner (removing impurities and drying in the shade) at the Institute of Highland Forest Science, Chinese Academy of Forestry, State Key Laboratory of Efficient Production of Forest Resources (25°45′9.44′′ N, 102°45′23.7′′ E), and stored at 25°C for drought resistance experiments.

In this study, ≥100 effective seeds of each tree species were screened by floating test, and hard-shelled seeds were mechanically shelled (*Q. franchetii*) or pretreated with concentrated sulfuric acid (*O. schwerinae*) (Yang et al., 2024a). PEG-6000 was used to simulate drought gradient (0-15%) (Yang et al., 2024b), and germination experiments were carried out in a sterilized culture system (25°C/75%RH/12h light). The germination potential (15d) and germination rate (30d) were measured, and the Duncan test was used to analyze the stress effect (P<0.05). The probability of suitable areas for the four tree species predicted by MaxEnt was combined with the laboratory drought adaptation data to verify the accuracy of the model in screening drought-resistant tree species.

The climate response prediction of the MaxEnt model and the tree species germination experiment may show the following corresponding relationship: (1) When the model predicts that the suitable growth zone will expand, the tree species germination rate in the simulated drought group should be significantly higher than that in the control group; (2) When the model predicts that the suitable growth zone will shrink, the germination rate in the control group should be higher than that in the simulated drought group; (3) If the model predicts that the suitable growth zone remains stable, there should be no significant difference in the germination rates between the two groups.

## 3 Result

### 3.1 Verify model optimization and accuracy

The optimal parameters of the four native tree species are inconsistent with the default MaxEnt model settings (RM=1 and FC=LQHPT). Compared with the MaxEnt model using default parameters, the optimized model has better fit and transferability, using the optimized model can reduce overfitting of the native tree species distribution model. Through the kuenm package analysis, all 1,160 models of the four native tree species were statistically significant. The optimal model was selected based on the omission rate of less than 5% and the minimum delta AICc value. The specific parameters are shown in **Supplementary Table S4**.

According to the optimal parameter setting, after 10 repetitions, the average AUC value of each crop was obtained. The AUC values of the four native tree species distribution models were all ≥ 0.960 (**Supplementary Table S1**), indicating that the optimized MaxEnt model effectively simulated the distribution of native tree species and had high accuracy and effectiveness in predicting the suitability of different native tree species.

### 3.2 The influence of environmental factors on the distribution of native tree species

The percentage contribution and permutation importance of each environmental factor to the distribution of suitable habitats of native tree species simulated by the MaxEnt model are shown in **Supplementary Table S1**. BIO3, BIO4, BIO6, BIO7, BIO11, BIO12 and BIO19 are the main variables affecting the distribution of native tree species in the dry-hot river valleys, and play a decisive role in the geographical distribution pattern of the four native tree species in the dry-hot river valleys of Yunnan. The main environmental factors affecting the distribution of *O. schwerina*e are BIO3 (19.6%), BIO4 (33.7%), BIO6 (26.5%) and BIO12 (17.2%); the main environmental factors affecting the distribution of *P. emblica* are BIO7 (61.3%) and BIO11 (37.3%); the main environmental factors affecting the distribution of *Q. franchetii* are BIO4 (56.3%), BIO6 (23.6%) and BIO19 (15.9%); the main environmental factors affecting the distribution of *S delavayi* are BIO4 (48.0%), BIO6 (31.5%) and BIO19 (11.1%). For the other corresponding variables of each native tree species, the climate contribution rate is less than 7%, which has little impact on the distribution of native tree species.

The Jackknife test results of environmental variables in **Figure 2b** show that the climate factors have the greatest impact on the regularized training gain results of the MaxEnt model among the environmental factors of the four native tree species. Among them, BIO4 and BIO6 have the greatest impact on the modeling of *O. schwerinae*, *Q. franchetii* and *S delavayi*, and BIO7 and BIO11 have the greatest impact on the modeling of *P. emblica*. In summary, temperature climate factors (BIO3, BIO4, BIO6, BIO7, BIO11) and precipitation climate factors (BIO12, BIO19) are the dominant bioclimatic factors affecting the potential geographical distribution of contemporary dry-hot valleys native tree species.

### 3.3 Probability of suitable habitats and potential distribution center shifts of native tree species in the past, present and future

**Figure 5:**
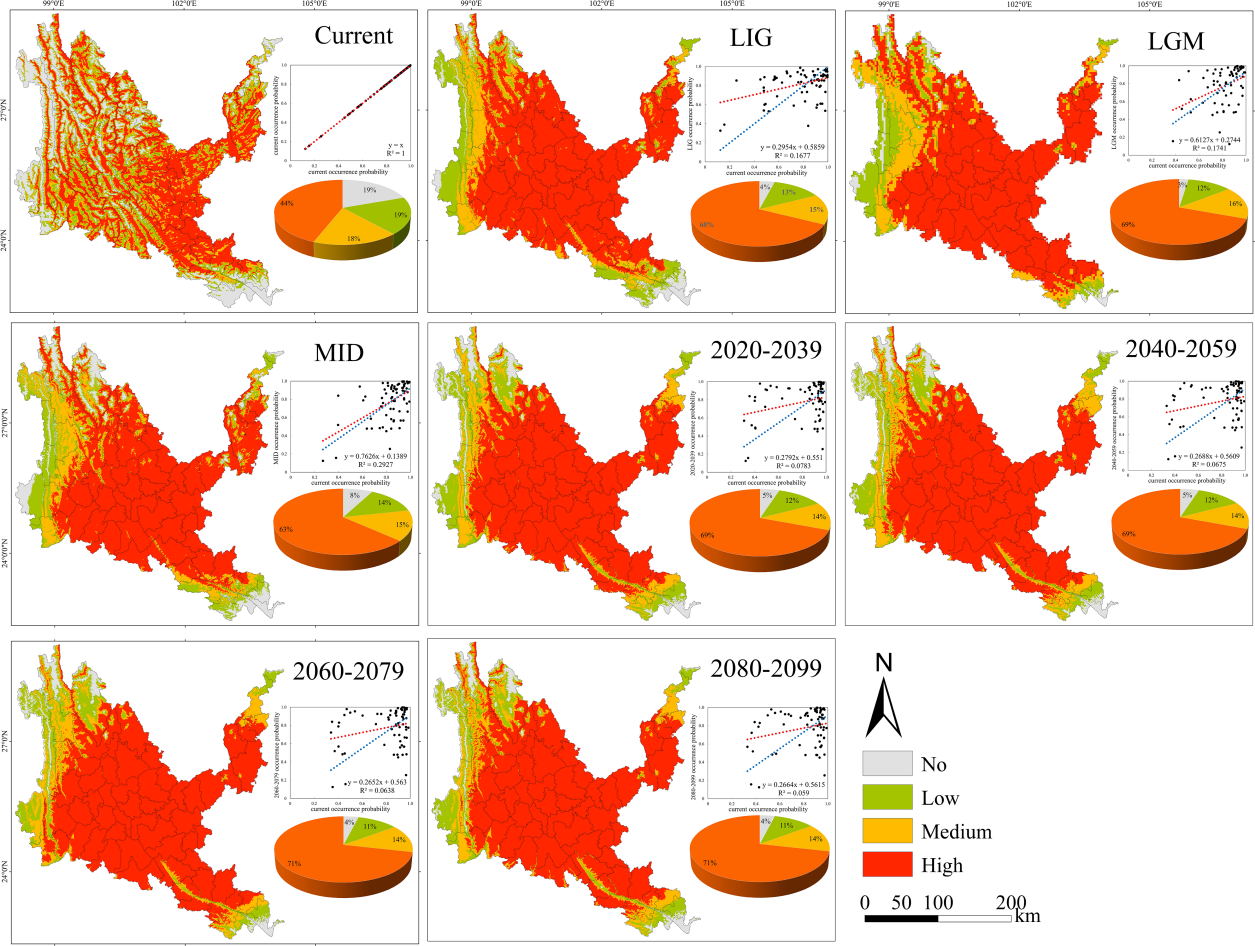
Geographical expression of the MaxEnt model of *Osteomeles schwerinae* calibrated under the current climate (upper panel). The predicted distribution of unsuitable, barely suitable, moderately suitable and highly suitable habitats on the different climate change scenarios (three time periods at four radiative forcing levels each) are provided below this map. The linear relationships between future and current occurrence probabilities and the statistical results of these analyses are showed within each of these panels (blue line: theoretical regression curve with *β_0_* = 0 and *β_1_* = 1; red line = empirical regression curve of each relationship). The pie chart sizes in the figure represent the area of no, low, medium, and high suitability areas as a percentage of the study area, respectively. The same below.

**Figure 6:**
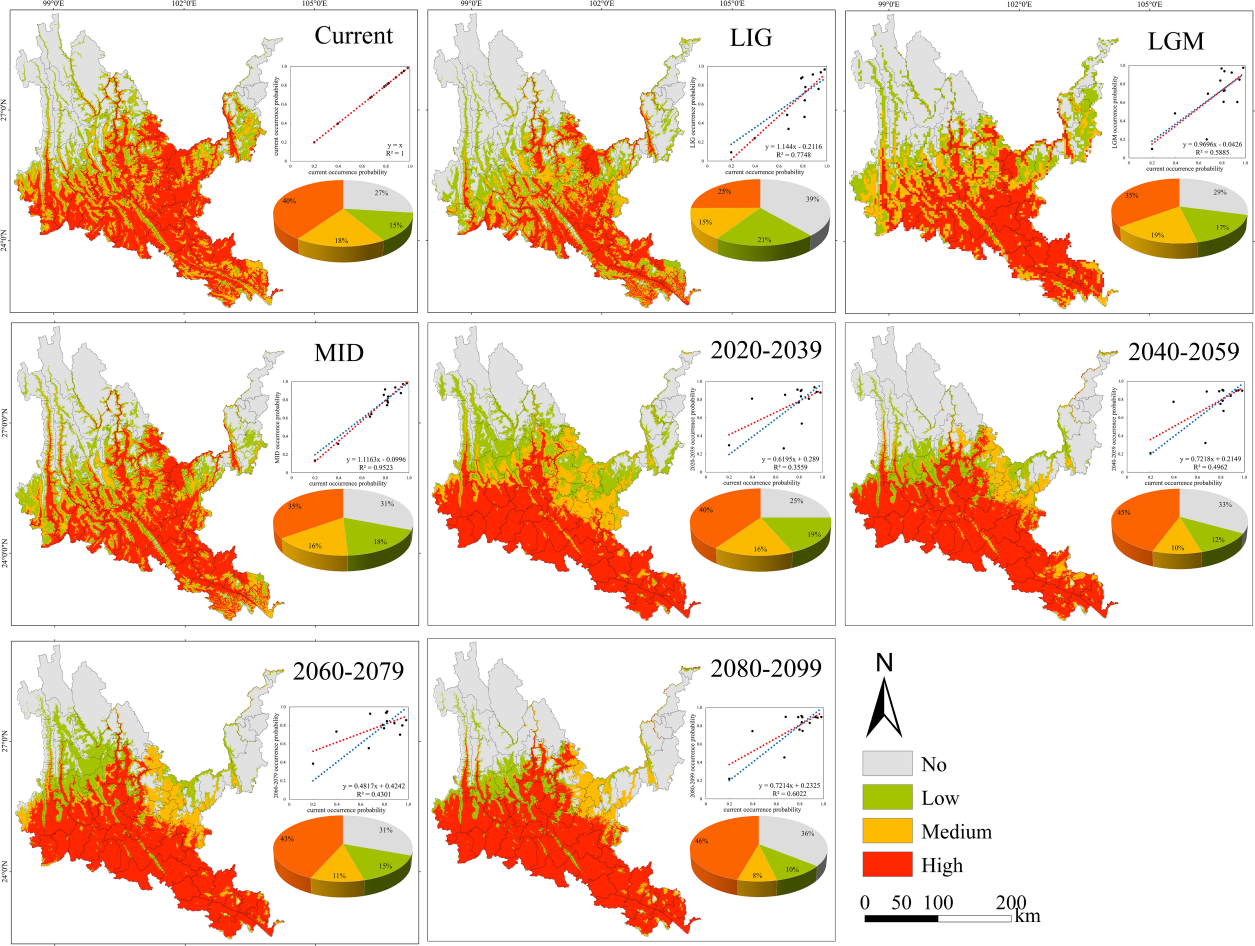
Geographical expression of the MaxEnt model of *Phyllanthus emblica* calibrated under the current climate (upper panel).

**Figure 7:**
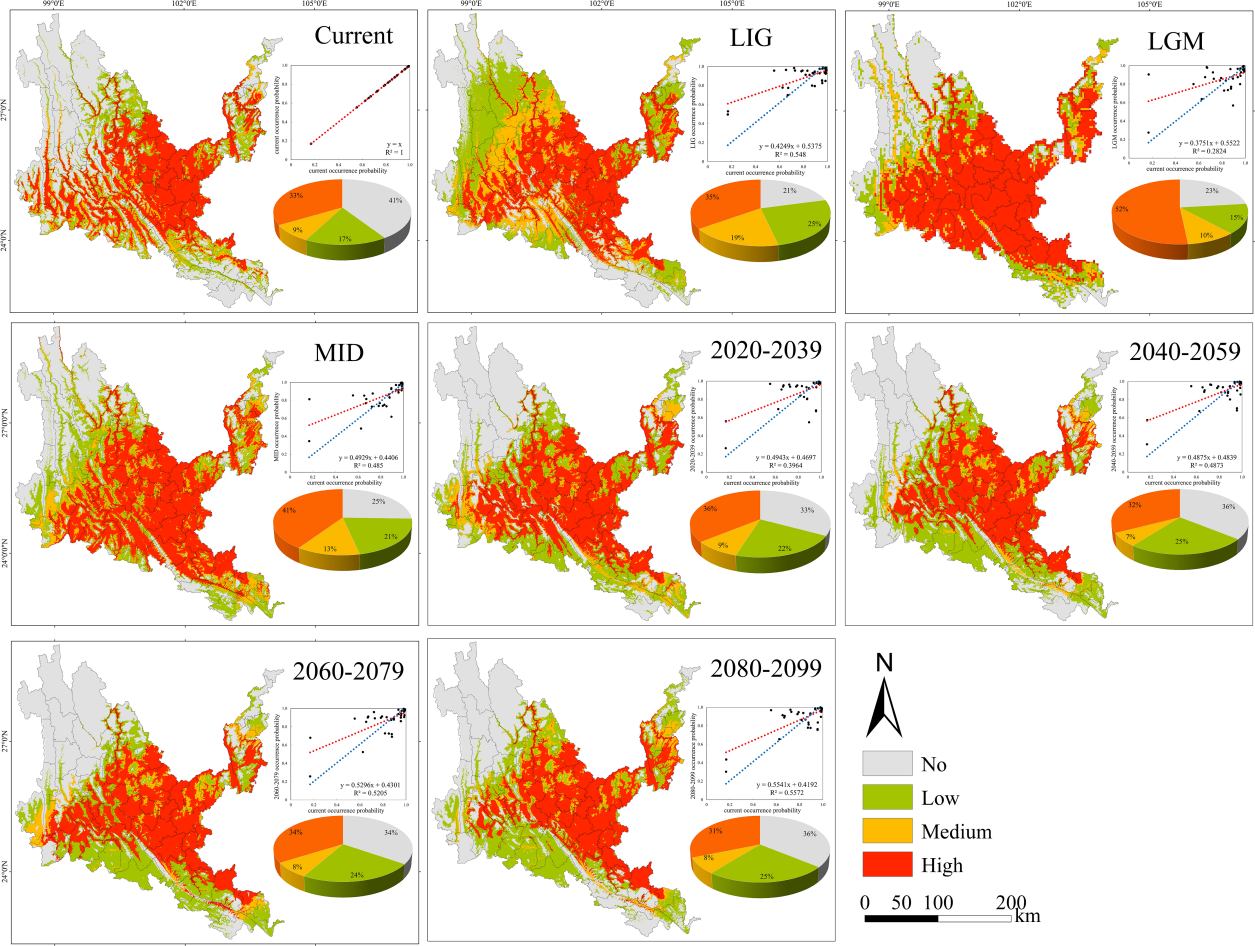
Geographical expression of the MaxEnt model of *Quercus franchetii* calibrated under the current climate (upper panel).

**Figure 8:**
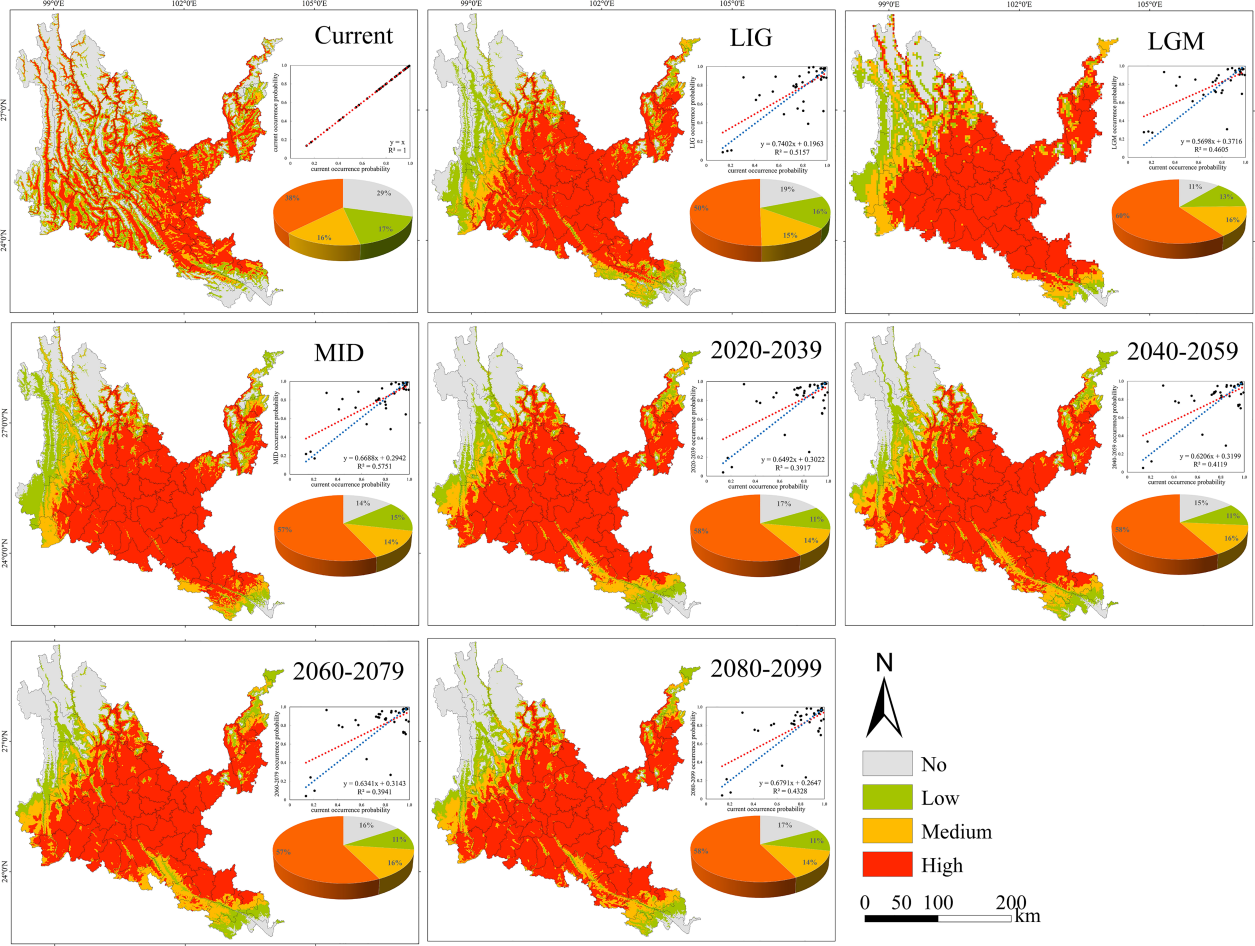
Geographical expression of the MaxEnt model of *Sapindus delavayi* calibrated under the current climate (upper panel).

The suitable habitats of the four native tree species are scattered under the contemporary climate, and are mainly distributed continuously or in strips along river valleys, mountains and rivers (**Figures 5**–**8**). The suitable habitats of *O. schwerinae* and *S. delavayi* basically cover the dry-hot valleys area, the suitable habitats of *P. emblica* are concentrated in the central and southern parts of the Dry-hot valleys area, and the suitable habitats of *Q. franchetii* are concentrated in the central part of the dry-hot valleys area. The total area of suitable habitats (low suitable habitat + medium suitable habitat + high suitable habitat) are all above 12.00×10^4^ km^2^ (**Supplementary Table S5**). Compared with the contemporary period, the areas of low and medium suitable habitats of *O. schwerinae* in the past three periods decreased, and the area of high suitable habitats increased; the areas of low suitable habitats of *P. emblica* and medium suitable habitats in the LGM period increased, and the areas of medium suitable habitats in the LIG and MID periods and high suitable habitats in the three periods decreased; except for the medium suitable habitat area in the LGM period, the area of suitable habitats of *Q. franchetii* increased in the other periods; the areas of low and medium suitable habitats of *S. delavayi* decreased, and the area of high suitable habitats increased. From the present to the future, the area of the *O. schwerinae* shows a trend of gradually increasing (2030s, 2050s, 2070s, 2090s), the total area of suitable habitats for *P. emblica* and *Q. franchetii* shows a bimodal trend of first increasing (2030s), then decreasing (2050s), increasing again (2070s) and decreasing again (2090s), and the total area of *S delavayi* habitat shows a unimodal trend of first increasing (2030s, 2050s) and then decreasing (2070s, 2090s).

**Figure 9:**
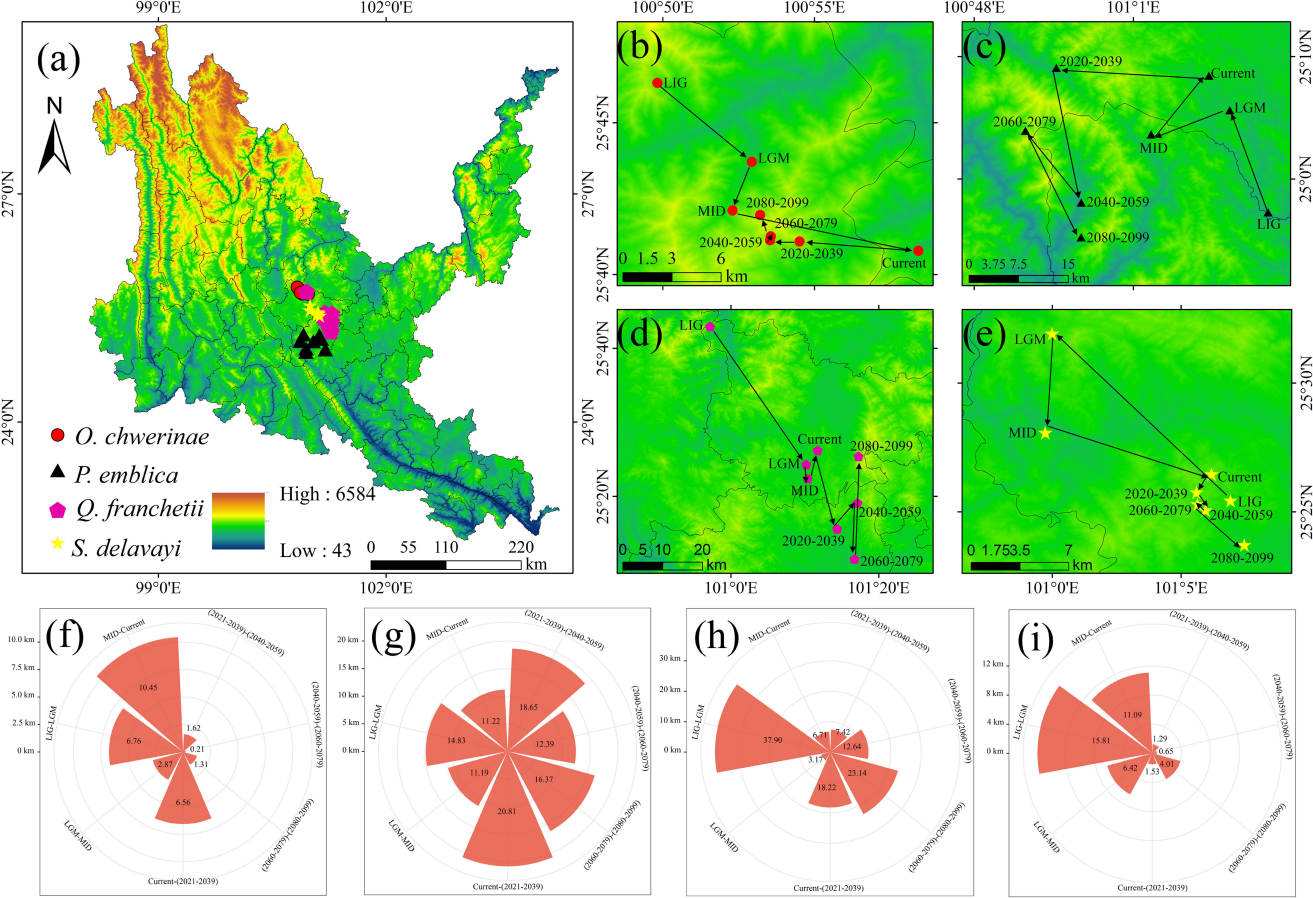
Centroid migration and area changes of four native tree species. **(a)** Centroid distribution map of four native tree species. **(b, f)** centroid distribution and migration distance of *Osteomeles schwerinae*. **(c, g)** centroid distribution and migration distance of *Phyllanthus emblica*. **(d, h)** centroid distribution and migration distance of *Quercus franchetii*. **(e, i)** Centroid distribution and migration distance of soapberry from *Sapindus delavayi*.

As can be seen from **Figure 9**, the past and future periods of *O. schwerinae* are biased to the northwest compared with the contemporary centroid, among which MID-Current is the farthest from other periods; the past period of *P. emblica* was biased to the south of the contemporary centroid, and the future period is biased to the southwest compared with the contemporary centroid, and the Current-2030s has the farthest migration distance; the past period of *Q. franchetii* was biased to the west compared with the contemporary centroid, and the future period is biased to the east compared with the contemporary centroid, and the migration distance is the farthest during the LIG-LGM period; the past period of *S. delavayi* was biased to the northwest compared with the contemporary centroid, and the future period is biased to the south compared with the contemporary centroid, and the migration distance is the farthest during the LIG-LGM period.

Under all climate change scenarios, the predicted probabilities of the past, future and current suitable habitats of the four tree species are positively correlated (**Figures 5**–**8**, **Supplementary Table S6**). Under all past and future climate scenarios, the values of β0 and β1 are different from the theoretical values under the contemporary climate scenario. Only the β0 of *P. emblica* in the past period (LIG, LGM, MID) is negative, predicting an increase in the area of the suitable habitat of *P. emblica* from the past period to the contemporary period, that is, the probability of *P. emblica* occupying the current unsuitable habitat and low suitable habitats decreases, and the probability of occupying the medium suitable habitat and high suitable habitats increases. However, the area of the suitable habitat of *O. schwerinae*, *Q. franchetii* and *S. delavayi* decreased from the past period to the contemporary period, and the area of the suitable habitat will increase in the future period.

### 3.4 Spatial variation patterns of four native tree species model validation framework

**Figure 10:**
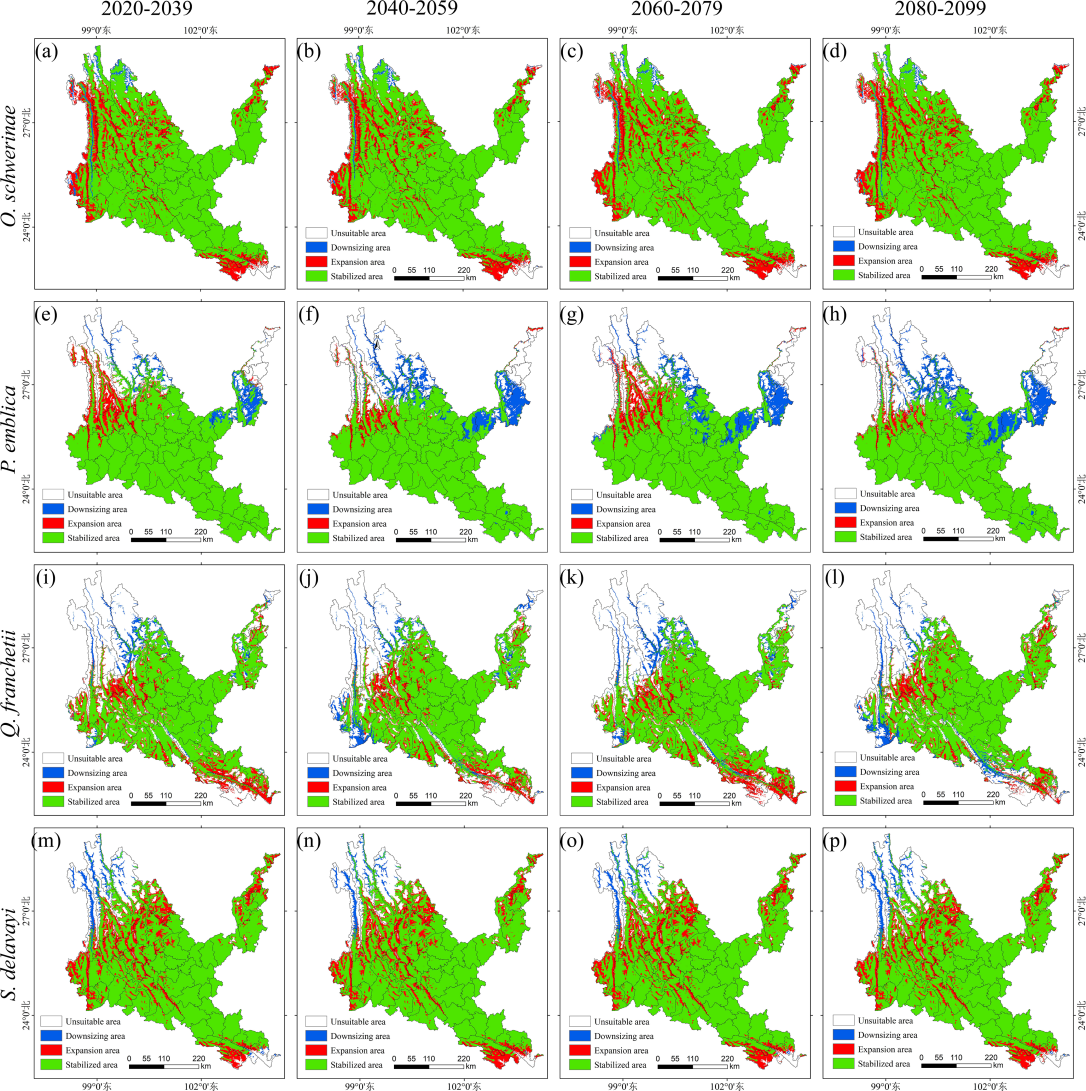
Suitable distribution changes of native tree species under different climate change scenarios. **(a–d)** *Osteomeles schwerinae*. **(e–h)** *Phyllanthus emblica*. **(i–l)** *Quercus franchetii*. **(m–p)** *Sapindus delavayi*. **(a, e, i, m)** 2030s, 2020-2039. **(b, f, j, n)** 2050s, 2040-2059. **(c, g, k, o)** 2070s, 2060-2079. **(d, h, l, p)** 2090s, 2080-2099.

The average rate of increase of *O. schwerinae* under the four climate scenarios is 16.50 (± 0.26)%, and the average rate of loss is 1.43 (± 0.23)%. The northwest of the new area is concentrated in the Hengduan Mountains and Gaoligong Mountains around dry-hot valleys of Nujiang, Lancangjiang and Jinshajiang. The loss area is distributed in strips along the Nujiang River, with a small amount distributed in Tengchong City, Deqin County and Shangri-La City. The unsuitable area for *P. emblica* in the future accounts for 1/5 of the entire dry-hot valleys. The new area is the largest in the two climate scenarios of 2030s and 2070s, with an increase rate of 6.67% and 6.09%, and the loss area is the largest in 2090s, with a loss rate of 11.93%; the stable area is concentrated in the central and southern parts of dry-hot valleys, the new area is located in the northwest of dry-hot valleys along the Nujiang and Lancangjiang rivers, and the loss area is located in the northwest and northeast of dry-hot valleys along the Jinsha River and Lancang River. Strip and block distribution of dry-hot valleys. The unsuitable area of *Q. franchetii* in the future accounts for 1/3 of the entire dry-hot valleys area. The newly added area under the four climate scenarios is basically the same, with an increase rate of 10.70 (± 0.68)%. The largest area will be lost in 2090s, with a loss rate of 5.36%; the stable area is located in the central, southern and northeastern regions of the dry-hot valleys, the newly added area is mainly inlaid in blocks in the western and southwestern regions of the dry-hot valleys, and the lost area is mainly distributed in strips along the dry-hot valleys of Jinsha River, Nujiang River, Lancang River and Yuanjiang River. The unsuitable area, stable area, newly added area and lost area of *S. delavayi* in Sichuan and Yunnan are stable, with an increase rate of 15.75 (± 0.55)%, and a loss rate of 2.64 (± 0.34)%; the newly added area is inlaid in blocks along the upper reaches of Jinsha River, Nujiang River and Lancang River in the stable area, and a small amount of blocks are distributed in the dry-hot valleys of the lower reaches of Yuanjiang River and Jinsha River in the southeastern and northeastern regions. The lost area is mainly located in the Hengduan Mountains in the northwest of the dry-hot valleys. In addition, we found that *O. schwerinae*, *P. emblica*, *Q. franchetii* and *S. delavayi* have a high degree of overlap in suitable areas of Yunnan’s dry-hot valleys and key ecological restoration areas in Yunnan Province (such as the Dianchi Lake Basin and the Jinsha River Basin), making them suitable as preferred tree species for ecological restoration projects (**Supplementary Table S7**, **Figure 10**).

### 3.5 Results of InVEST model

**Figure 11:**
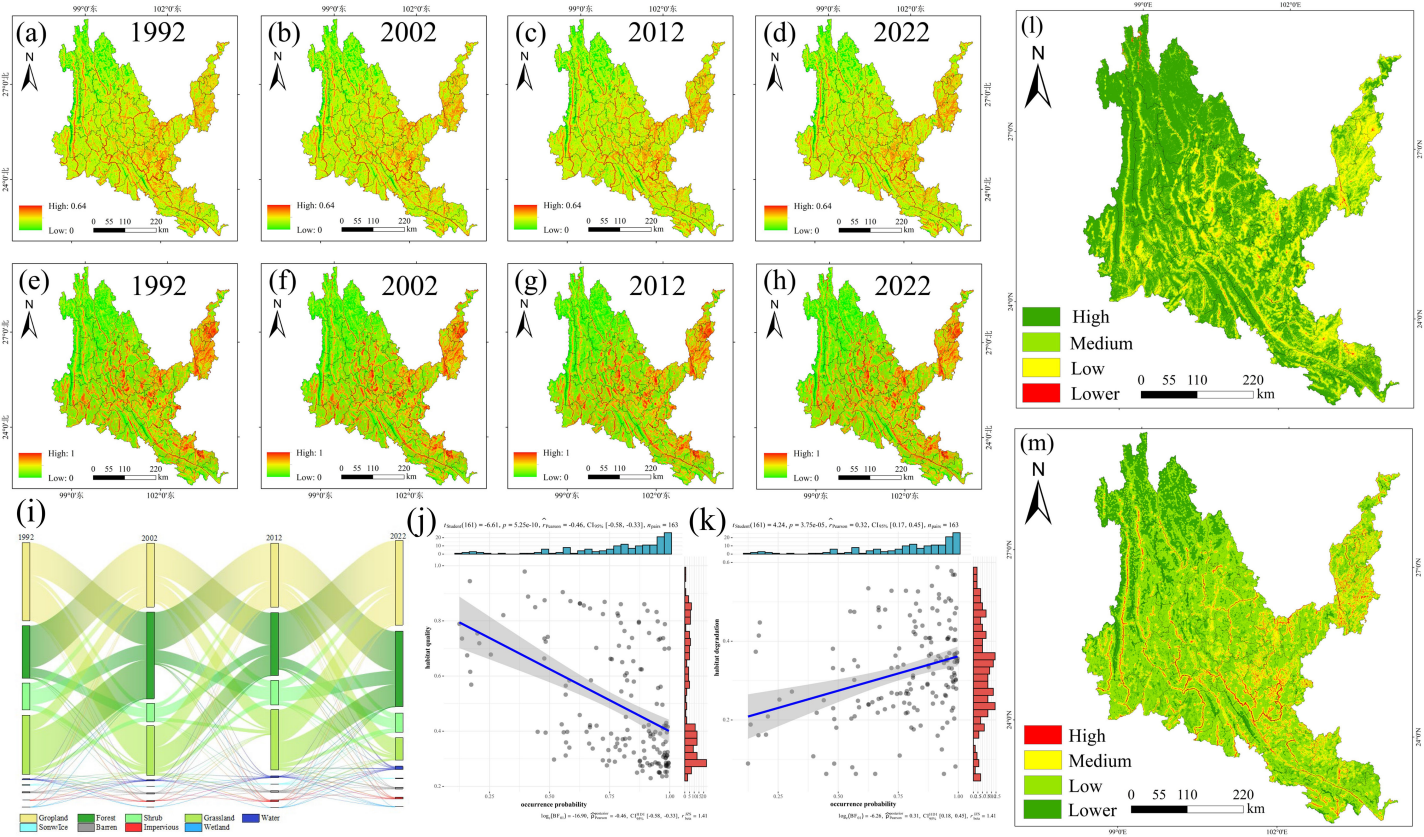
Results of InVEST model. **(a-d)** Habitat degradation in each period from 1992 to 2022. **(e-h)** Habitat quality in each period from 1992 to 2022. **(i)** Sankey diagram of land-use transfer in dry-hot valleys of Yunnan from 1990 to 2022. **(j)** Habitat degradation and occurrence Point relationship diagram. **(k)** Habitat quality and occurrence point relationship diagram. **(l)** distribution area of each category of Habitat degradation after superposition of multiple periods. **(m)** distribution area of each category of Habitat quality after superposition of multiple periods.

As shown in **Supplementary Table S8**, the cropland area first decreased in 2002, and then showed a trend of continuous increase, the forest area continued to increase, the shrub area fluctuated, the grassland area continued to decrease, and the water and impervious area increased. During 1992-2022, the land use transfer in the dry-hot valleys region was mainly concentrated in the following land types: cropland, grassland, forest, shrub and impervious. It was mainly manifested in the increase of cropland, forest and impervious, and the decrease of shrub and grassland. The main feature was the mutual conversion between cropland, grassland, forest and shrub. Cropland was mainly converted into forest and grassland, forest was mainly converted into cropland and shrub, and shrub was mainly converted into cropland, forest and grassland. In addition, the urbanization process in the dry-hot valleys region was mainly to expand the impervious land area at the expense of cropland and grassland. The probability of occurrence of native tree species is positively proportional to the degree of habitat degradation and inversely proportional to the habitat quality. The occurrence points of native tree species are mostly concentrated in areas with a habitat degradation degree of 0.3-06 and a habitat quality of 0.2-0.4 (**Figure 11**).

### 3.6 Regional division of native tree species based on InVEST-MaxEnt coupling model

**Figure 12:**
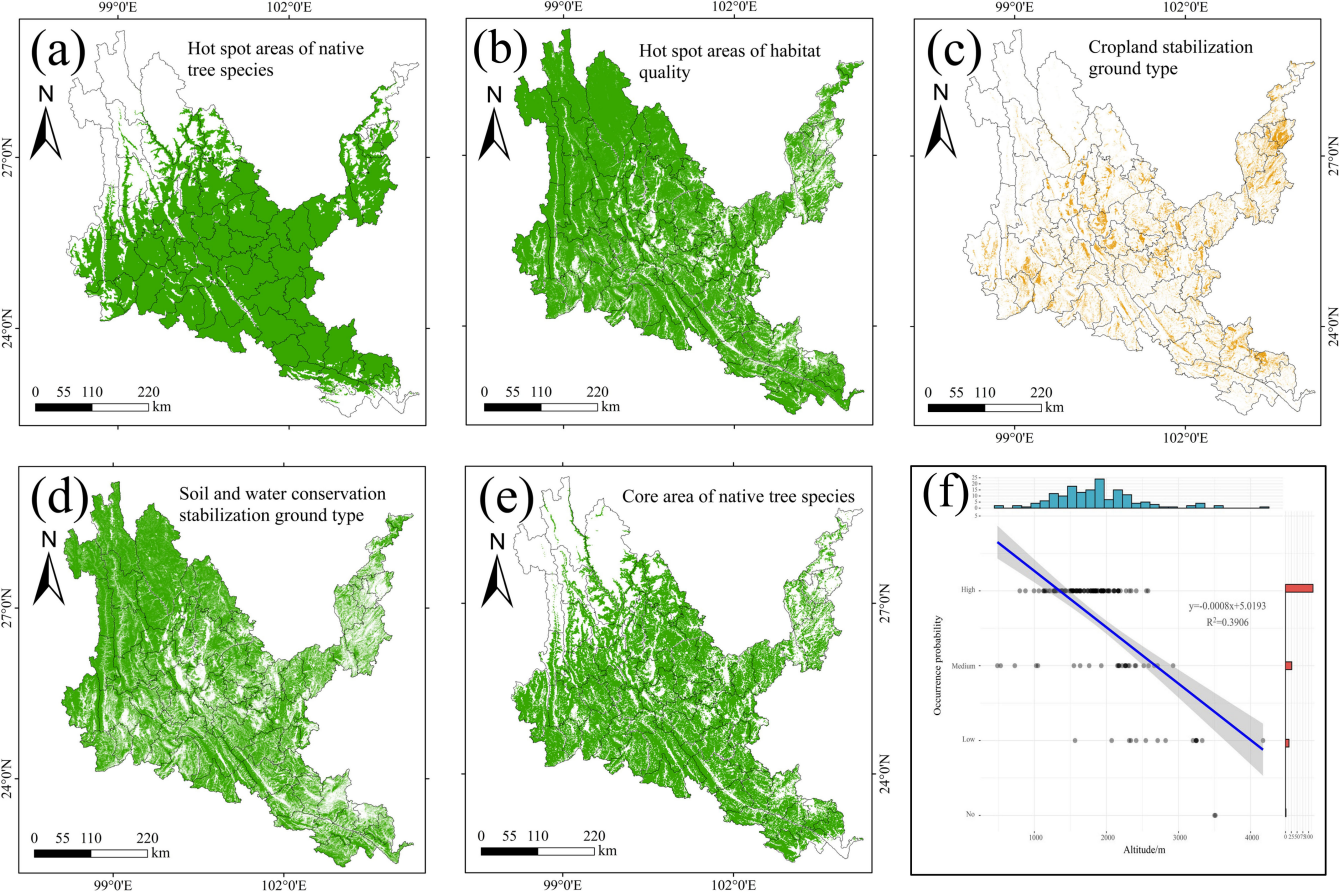
Results of coupled InVEST-MaxEnt. **(a)** The core area of native tree species. **(b)** Hot spot areas of habitat quality. **(c)** the stable cultivated land area. **(d)** the stable soil and water conservation area. **(e)** the core protection area of native tree species. **(f)** Map of the relationship between the occurrence points of native tree species and altitude.

As shown in **Figure 12**, the hotspots of native tree species are mainly distributed in dry-hot valleys, mainly concentrated in the middle, southeastern and northeastern parts of dry-hot valleys, and the blank areas are mainly in the northwest, Yuanyang County, Jinping County and Pingbian County in the southeast, and Yongshan County and Suijiang County in the northeast. The hotspots of habitat quality basically cover the entire dry-hot valleys, and the blank areas are scattered or densely distributed in the urban agglomeration area in the middle of dry-hot valleys and the karst areas in the northeast and southeast. The stable cultivated land types are mainly scattered and strip-shaped in the middle, southeastern and northeastern parts of dry-hot valleys, which also shows that such areas are areas with high human activities and high residential clusters. The stable soil and water conservation area mainly covers the entire dry and hot valley area in the form of wrapped cultivated land. After superimposing the hotspots of native tree species and the hotspots of habitat quality, it is defined as the core area of native tree species, which is mainly distributed in the middle, southeastern and northeastern parts of dry-hot valleys. There are many block blank areas in the middle, southeastern and northeastern parts, mainly cultivated land and residential areas. The probability of occurrence points of native tree species is inversely proportional to the altitude of dry-hot valleys, that is, the higher the altitude, the fewer the occurrence points of native tree species. The occurrence points of native tree species are mainly located between 1,000-2,500m above sea level in dry-hot valleys.

**Figure 13:**
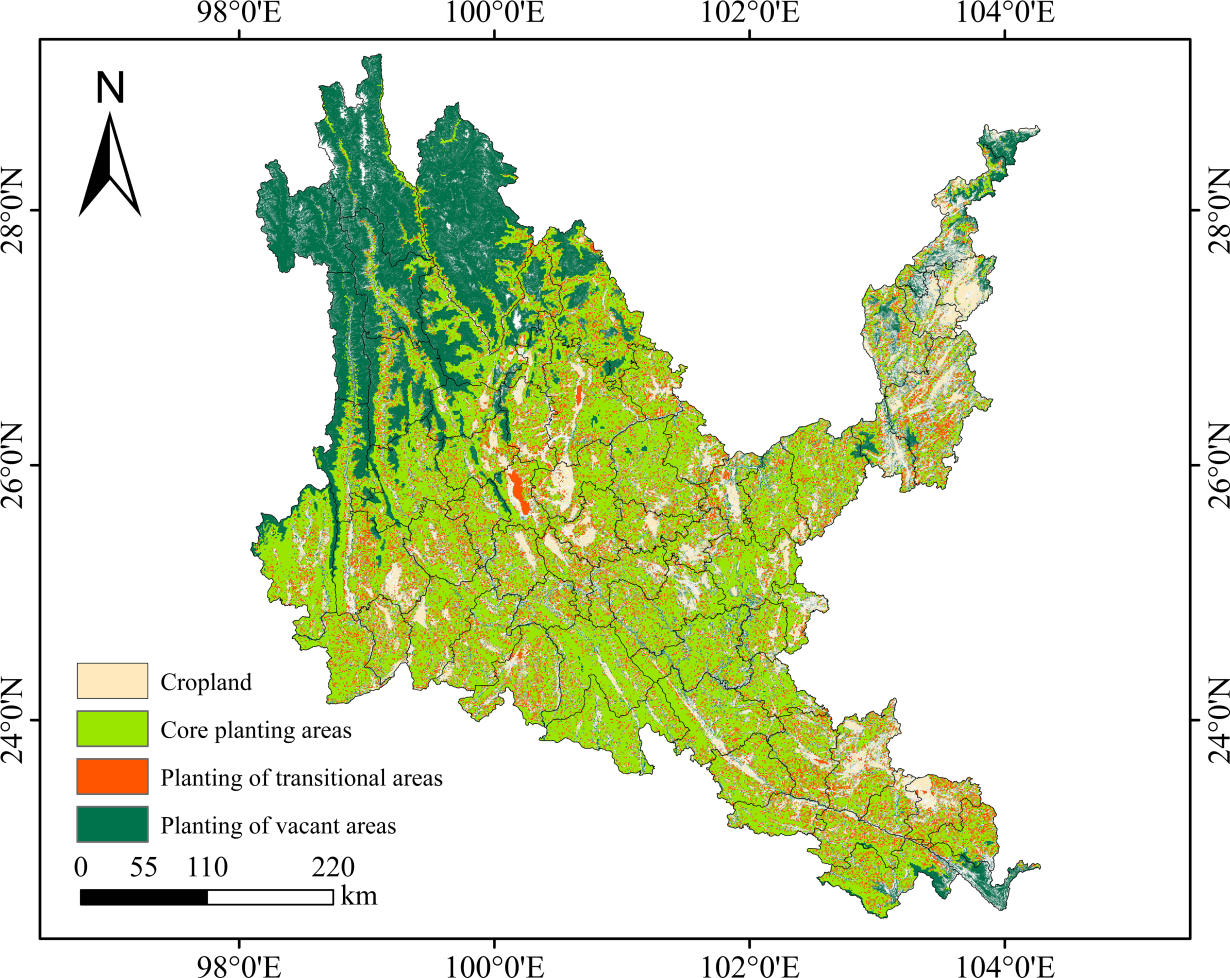
Suitability mapping of native tree species in dry-hot valleys of Yunnan.

The areas of stable cultivated land and stable soil and water conservation land in dry-hot valleys are 19,960.63 km^2^ and 135,104.30 km^2^ (**Supplementary Table S9**), accounting for 9.72% and 65.80% of the area of dry-hot valleys, respectively. The core planting area is 91,307.54 km^2^, and the core area of native tree species in dry-hot valleys is 119,845.36 km^2^. The difference between the two areas is 28,537.82 km^2^ of the core transition area, and 43,796.76 km^2^ of the planting blank area, which is the difference between the land area of stable soil and water conservation area and the core planting area. As shown in **Figure 13**, the core planting area in dry-hot valleys is mainly located in the central, southeastern and northeastern regions of dry-hot valleys, with cultivated land densely embedded in them, and the planting transition area is densely distributed in the core planting area in a scattered manner, and only in the Dali city area is a block distribution. The blank areas for planting native tree species are mainly located in Deqin County, Gongshan County, Weixi County, Lanping County, Shangri-La City, Ninglang County, Yulong City and other areas in the northwest of dry-hot valleys, Jinping County, Hekou County and Pingbian County in the southeast, and Luquan County, Dongchuan District, Qiaojia County, Zhaoyang District, Yongshen County and Suijiang County in the northeast. By comparing with the land and space planning data of Yunnan Province, it is found that the core vegetation restoration areas identified in this study are highly consistent with the “degraded land restoration priority areas” planned by the government. The research results can be used to optimize the vegetation restoration strategy within the ecological protection red line area of Yunnan Province, improve the forest carbon sequestration capacity, and be consistent with the national “dual carbon” goals.

### 3.7 Field survey and seed germination experiment of native tree species

**Figure 14:**
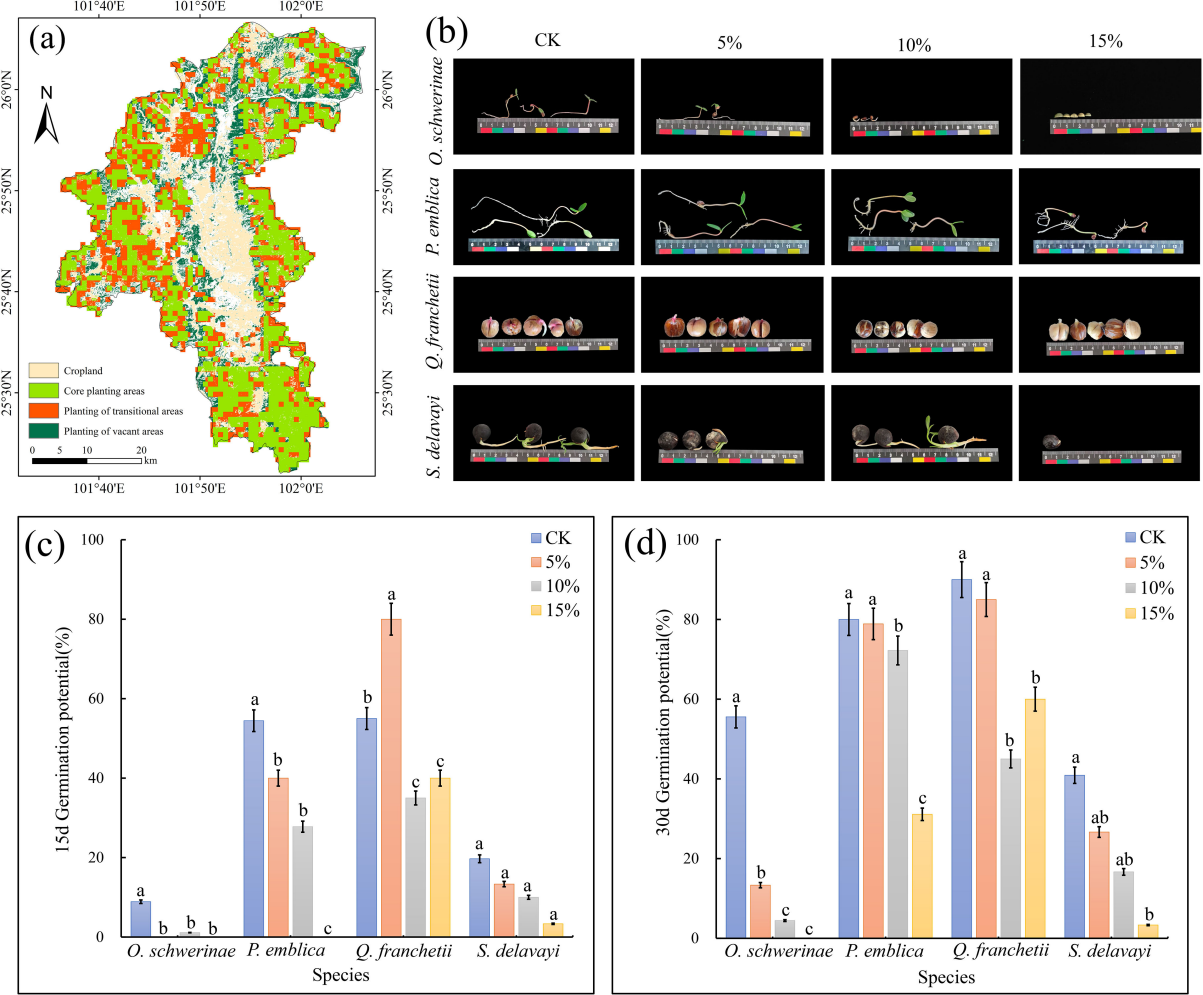
Field survey and seed germination experiment of native tree species. **(a)** Suitability mapping of native tree species in Yuanmou. **(b)** Photos of seed germination of native tree species in various treatments. **(c)** seed germination potential. **(d)** seed germination rate. Error bars in the figure indicate 95% confidence intervals. The different lowercase letters indicate significant differences between treatments (p < 0.05).

The actual survey results show (**Supplementary Table S10**) that the four native tree species can be distributed in Yuanmou County, and their actual distribution area reaches 158.25 km^2^. Among them, Quercus contorta has the largest actual distribution area, which is 94.45 km^2^, and the actual distribution area of Sapindus mukorossi is the smallest, which is only 0.11 km^2^. The four native tree species are mainly distributed in Xinhua, Yangjie, Jiangyi, Laocheng, Wumao, Yuanma, Liangshan and Jiangbian in Yuanmou County. The cultivated land area of Yuanmou County is mainly located in the central and northern parts of the county. The planting core area and planting transition area mainly cover the surrounding areas around the cultivated land, and the planting blank area is located in the northern part of the county (**Figure 14a**).

Under simulated drought conditions, the germination potential and germination rate of *O. schwerinae*, *P. emblica* and *S. delavayi* all showed a decreasing trend with increasing PEG concentration, while the germination potential of *Q. franchetii* was the highest at 5% PEG and the germination rate was the lowest at 10% PEG. We constructed 100 random points in Yuanmou County and extracted the probability of occurrence of random points under contemporary and future climate scenarios. It can be seen that the MaxEnt of *O. schwerinae* predicts that the probability of this tree species occupying the suitable area in Yuanmou County will increase (Current=0.8265(± 0.19) vs.Future=0.9865(± 0.0056); the difference between the current and future probability of occupying the suitable area=-0.16); the MaxEnt of *P. emblica* predicts that the probability of this tree species occupying the suitable area in Yuanmou County will decrease (Current=0.9015(± 0.1619)vs.Future=0.4099 (± 0.026); the difference between the current and future probability of occupying the suitable area=-0.4916); the MaxEnt of *Q. franchetii* predicts that the probability of this tree species occupying the suitable area in Yuanmou County will increase slightly (Current=0.9533(± 0.12)vs.Future=0.9616 (± 0.0012); The difference between the current and future probability of occupying the suitable area = -0.0083); MaxEnt of *S. delavayi* predicts that the probability of this tree species occupying the suitable area in Yuanmou County will increase (Current = 0.8699 (± 0.15) vs. Future = 0.9736 (± 0.0054); The difference between the current and future probability of occupying the suitable area = -0.1037).

The 15-day germination rates of the four tree species were analyzed, and only *P. emblica* and *Q. franchetii* corresponded to the predicted results. The results of the seed germination experiment showed that the germination rate of *P. emblica* in the 5% PEG drought stress simulation group was lower than that of the control group. For *Q. franchetii*, the germination rate of the 5% PEG drought stress simulation group was higher than that of the control group. As time and drought intensity intensified, the germination rates of the four native tree species showed a decreasing trend. The root length and seedling length of the seeds of *O. schwerinae* and *Q. franchetii* gradually shortened with the increase of PEG concentration, and the cotyledons became smaller and disappeared. Although the germination rates of *P. emblica* and *S. delavayi* in the PEG drought stress simulation group were lower than those of the control group, their seedling length and root length at higher PEG concentrations were similar to those of the control group, which further reflected that these two seeds had a high tolerance to drought climate (**Figures 14b–d**).

## 4 Discussion

### 4.1 Importance of MaxEnt model optimization

The MaxEnt model is based on a machine learning algorithm. In a large number of studies, the MaxEnt model has been verified as one of the most extensive and accurate models for predicting species distribution (Phillips et al., 2006; Boral and Moktan, 2021; Li et al., 2023). In this study, the optimized MaxEnt model was coupled with the InVEST model to divide the core planting areas, planting transition areas, and planting blank areas of four native tree species in the dry-hot valleys, and the suitability of the native tree species was analyzed. However, the MaxEnt model is known to be sensitive to sampling bias (non-random sampling) and prone to overfitting, which affects the transferability of the model. However, these issues have not received enough attention in previous studies. The default MaxEnt model parameters may not be suitable for all species, and we found that most studies used default parameters to simulate species distributions, which led to overfitting and may reduce the accuracy of the results or produce unexplainable results (Fourcade et al., 2014; Halvorsen et al., 2016). In order to improve the performance of the MaxEnt model, this study uses the “kuenm” R package to optimize the two parameters of feature combination(FC) and regularization multiplier(RM) to help reduce model overfitting and complexity, thereby significantly improving the model’s performance. prediction accuracy (Zeng et al., 2016; Radosavljevic and Anderson, 2014). From all 1,160 model species selected from the four native tree species, the optimal model with a missing rate of less than 5% and a minimum delta AICc value was screened. The results showed that the AUC values of the four native tree species distribution models were all ≥0.960 (**Supplementary Table S1**), indicating that the optimized model has low complexity, high transferability and prediction accuracy, and can be used to predict the distribution of four native tree species in hot-dry valley. This paper analyzes and screens the environmental variables used, thus solving the problem of inaccurate modeling results caused by strong multicollinearity between factors.

### 4.2 Importance of environmental factors

Among the corresponding variables of the four native tree species simulated in this study, temperature climate factors (BIO3, BIO4, BIO6, BIO7, BIO11) and precipitation climate factors (BIO12, BIO19) are the dominant bioclimatic factors affecting the potential geographical distribution of contemporary dry-hot valleys native tree species, among which temperature is more important than precipitation. In the study of simulating the ecological suitability zoning of *Genus Sapindus* (Liu et al., 2021) and *P. emblica* (Li et al., 2021), the main environmental factors affecting the distribution of *S. delavayi* were precipitation in the warmest season (contribution rate of 37.9%) and average temperature in the coldest season (contribution rate of 15.9%), and the main environmental factors affecting the distribution of *P. emblica* were 9 environmental factors related to temperature. The environmental factors that affect the distribution of *S. delavayi* and *P. emblica* are dominated by temperature. The contribution rate of other environmental factors is relatively low (less than 7%). They directly or indirectly affect temperature and precipitation to change the species distribution pattern and play a compensatory role in the distribution of native tree species. The obvious dry and wet seasons, the imbalance of water and heat ratio, high heat, low precipitation and strong evaporation make the valley and high mountain drought more strongly interfere with the distribution of native tree species than the high temperature environment. Adequate precipitation and stable temperature are the basic prerequisites for the survival of native tree species in dry-hot valleys. In this area, the water demand of plants increases greatly to maintain plant transpiration and photosynthesis. This is also a major factor restricting the growth and distribution of native tree species in dry-hot valleys (Stockwell, 1999).

After checking the flowering and fruiting periods of native tree species, it was found that most of the flowering periods are from March to June, and the fruiting periods are from September to November. Improper drought and water shortage will destroy the normal operating mechanism of the plant (Zinta et al., 2016). Seasonal drought and reduced precipitation in this area directly affect the seed setting, germination and seedling growth of native tree species, prevent the reproduction of native tree species, and even cause plant death. This is an important factor leading to differences in the geographical spatial distribution of native tree species. The interaction between climate change and human activities has a significant impact on the vegetation of hot-dry valleys. Global climate warming has led to rising temperatures, changes in precipitation patterns, and an increase in extreme weather events. These changes have had a negative impact on the growth and adaptability of vegetation in dry-hot valleys. Challenges, human activities such as greenhouse gas emissions and deforestation have exacerbated the speed and magnitude of climate change, further exacerbating the vulnerability and vulnerability of vegetation in dry-hot valleys (Yin et al., 2022). However, we innovatively introduced the InVEST model to define the habitat quality of dry-hot valleys based on land use data, which to some extent weakened the impact of Yunnan’s complex terrain on the MaxEnt model simulation, which is consistent with the actual situation of dry-hot valleys. When using the MaxEnt model to predict the suitable habitats of potential suitable tree species, this experiment has screened the sample points and environmental factors, but there are still some deviations between the predicted distribution areas of a small number of tree species and the actual distribution areas in the MaxEnt model prediction results. This difference may be due to the failure to consider factors such as population competition, natural regeneration capacity, natural disasters, pests and diseases, and local microclimate (Ma and Li, 2023). In the future, in order to further improve the accuracy and reliability of species distribution models, more factors need to be considered, such as coupling MaxEnt with individual models, incorporating interspecific competition coefficients and resource allocation equations (dynamic model integration); using high-resolution data (above 10m) to extract vegetation aggregation index, neighborhood competition pressure, etc., and construct spatially explicit biotic interaction factors (high-resolution biotic interaction layer); quantifying the regulatory effect of pollinator-plant interaction networks on the stability of suitable habitats based on food web databases (multi-trophic level modeling).

### 4.3 Spatial distribution pattern and centroid migration of suitable areas for native tree species

Understanding the changes in the potential distribution pattern of species under the background of climate change is crucial for measuring the impact of climate change on species and formulating conservation strategies to maintain ecological balance (Deb et al., 2017). Climate is the most important factor in determining the survival and distribution of species, and the distribution characteristics of plants are the most direct response to climate. Based on the MaxEnt model, this study simulated the potential geographical distribution of four native tree species in dry-hot valleys of Yunnan at different times, revealing that the suitable habitats of *O. schwerinae* and *S. delavayi* basically cover dry-hot valleys, the suitable habitats of *P. emblica* are concentrated in the central and southern parts of dry-hot valleys, and the suitable habitats of *Q. franchetii* are concentrated in the central part of dry-hot valleys, which are basically consistent with the conclusions of the field survey (Li and Zhao, 2007; Liu et al., 2012). This also indirectly proves that the optimized MaxEnt model has high simulation accuracy and good effect for the four native tree species in dry-hot valleys. From the distribution map of geographical suitable habitats, it can be observed that compared with the past three periods, the low and medium suitable habitats of *O. schwerinae* decreased, while the high suitable habitats increased; the low suitable habitats of *P. emblica* and the medium suitable habitats of LGM expanded, while the medium suitable habitats of LIG and MID and the high suitable habitats of the three periods decreased; except for the medium suitable habitats of LGM, the suitable habitats of *Q. franchetii* showed an expanding trend in other periods; the low and medium suitable habitats of *S. delavayi* decreased, while the high suitable habitats increased. It is inferred that the mountains and valleys embedded in the Qinghai-Tibet Plateau and the Yunnan-Guizhou Plateau were formed by the erosion and cutting of large rivers. Under the influence of the subtropical climate, the plateau valley landforms are backed by wind and rain shadow areas and valley lowlands, forming a typical dry and hot climate (Yang et al., 2016). Due to the special biological and physiological and ecological adaptation mechanisms of the native tree species in the dry-hot valleys, they are less affected even in the face of dry and hot climate environments.

In addition, during the last interglacial period, the last glacial maximum, and the middle Holocene, the suitable habitats of native tree species in dry-hot valleys were highly concentrated, while in modern times, the potential suitable habitat patches are severely fragmented, which is speculated to be related to the ecological fragmentation aggravated by the increase in energy consumption, CO_2_ emissions, logging, land reclamation and other human activities. In the next four periods, the area of suitable habitats of *O. schwerinae* will gradually increase, the total area of suitable habitats of *P. emblica* and *Q. franchetii* will show a bimodal trend of first increasing, then decreasing, then increasing, and then decreasing, and the total area of habitats of *S. delavayi* will show a unimodal trend of first increasing and then decreasing. Moreover, under all climate change scenarios, the predicted probabilities of the past, future and current suitable habitats of the four tree species are positively correlated. It can be inferred that the four native tree species can adapt well to future climate conditions. However, except for *O. schwerinae*, the suitable habitat areas of *P. emblica*, *Q. franchetii* and *S. delavayi* have all shown a downward trend over time. It is speculated that this may be due to the drastic and irregular changes in temperature and precipitation and the frequent extreme events under future climate conditions, which have a greater impact on these three native tree species and lead to increased habitat fragmentation (Thomas et al., 2004; Monteith et al., 2015). Therefore, we predict that under the background of global warming in the future, the three native tree species of *P. emblica*, *Q. franchetii* and *S. delavayi* will face survival risks.

The spatial distribution pattern of plants is very sensitive to climate change, and the distribution areas are very obvious in longitude and latitude. For example, tree species in low-latitude areas of Europe tend to expand to high latitudes, and tree species commonly distributed in high-latitude areas are losing their current distribution areas while few new potential distribution areas emerge (Dyderski et al., 2018). For the four typical native tree species in dry-hot valleys of Yunnan, they will obtain new suitable distribution areas through migration to make up for the loss of some existing suitable distribution areas, and the spatial position of the suitable distribution areas will change accordingly. Overall, under the future climate scenario, the concentration of the potential distribution areas of native tree species will increase, and the degree of fragmentation will decrease to varying degrees. The loss of distribution areas is mainly concentrated in the Hengduan Mountains in the northwest of the dry-hot valleys, the Gaoligong Mountains in the west, and the Yunnan-Guizhou Plateau in the northeast. The increase in areas is mainly expanding to the dry-hot valleys of Jinsha River, Nujiang River, Lancang River and Yuanjiang River, indicating that these areas may be sensitive areas for future pattern changes and should be paid attention to. In this experiment, the four native tree species will also spread in multiple directions under different climate scenarios, which is similar to the situation in the northwestern United States where tree and shrub groups spread to high latitudes and high altitudes while migrating in multiple directions (Shafer et al., 2001). The centroid of *O. schwerinae* generally showed a trend of first migrating to the southeast and then to the northwest; the centroid of *P. emblica* generally showed a trend of first migrating to the northwest and then to the south; the centroid of *Q. franchetii* generally showed a trend of first migrating to the southeast and then to the north; *S. delavayi* generally showed a trend of first migrating to the northwest and then to the southeast. Related studies have shown that under future climate scenarios, as the global climate warms, most animals and plants have a trend of moving to higher latitudes and higher altitudes (Fang et al., 2018). The centroids of the potential distribution areas predicted by the study have the same trend except for *S. delavayi*. In the future, the populations in the southeastern and central regions of the study area may have higher genetic diversity and genetic genes, and may have stronger adaptability to dry-hot valleys. With the further development of global warming, some tree species in the northern and northwestern regions of dry-hot valleys, especially those that are difficult to withstand drought stress, will face the risk of shrinking suitable habitats and reduced genetic diversity. However, for some heat-loving species, their suitable habitats are expected to expand further (Lyons et al., 2010; Hagen et al., 2007). Therefore, the four native tree species that are resistant to high temperature, drought, and barrenness will be the most important defenders of dry-hot valleys ecosystem. While fully considering the use of suitable tree species for ecological restoration and afforestation in the priority planting areas of dry-hot valleys and the introduction and cultivation of urban landscaping, factors such as climate-related instability, drought severity, and human interference must be considered, which may be the main determinants of the growth and death of native tree species in dry-hot valleys.

### 4.4 Regional division based on coupled InVEST-MaxEnt model

The Habitat Quality model was used to calculate the habitat degradation degree (**Figures 10a–d**) and habitat quality in dry-hot valleyss of Yunnan from 1992 to 2022. The low-degradation and high-quality habitats are mainly distributed in the soil and water conservation type areas. There are a large number of small patches of low-degradation and high-quality habitats scattered in the northwest and central parts of dry-hot valleys. The high-degradation and high-quality habitats are attached to the low-degradation and high-quality habitats, and are distributed in bands and fragments. Habitats with high degradation and low quality are mainly distributed in urban settlements and areas with large human activities on both sides of dry-hot valleys of the Jinsha River, Nu River, Lancang River and Yuan River. The patch area is large and the concentrated distribution is obvious. Habitat degradation and habitat quality are inversely proportional. From 1992 to 2002, the land use change in dry-hot valleys was small, the habitat degradation was small, and the habitat quality was high. From 2002 to 2012, urbanization progressed continuously, human activities increased significantly, land use changed greatly, habitat degradation was large, and habitat quality became low. From 2012 to 2022, with the layout and implementation of ecological and environmental protection measures, both ecological degradation and habitat quality have changed well. We found that in the past 20 years, the rate of increase in cultivated land area and the rate of degradation of soil and water conservation type areas have significantly accelerated. Woodlands, shrubs and grasslands are gradually being eroded by artificial cultivated land, and human activities have a great impact. This may be caused by the special terrain environment of the dry-hot valleys area leading to changes in agricultural climate resources. The area suitable for the cultivation of tropical economic crops in the region will increase. The reasonable layout of suitable tree species can improve carbon sequestration capacity. The results of this study can provide data support for the “Yunnan Province Carbon Neutral Afforestation Plan”.

In order to scientifically identify the core areas for the protection of native tree species and the blank areas for planting in dry-hot valleys and improve the efficiency of ecological restoration and afforestation in this area, the experimental coupling InVEST-MaxEnt model was studied to fully consider the potential distribution of native tree species under the synergistic effect of natural conditions and human interference conditions, and accurately identify the core areas for the protection of native tree species and the blank areas for planting in dry-hot valleys. We compared the probability of tree species occurrence points with the degree of habitat degradation and habitat quality, and found that the probability of native tree species occurrence points was positively proportional to the degree of habitat degradation and inversely proportional to the habitat quality. The occurrence points of native tree species were mostly concentrated in areas with a degree of habitat degradation of 0.3-06 and a habitat quality of 0.2-0.4. On the one hand, this area has a high population concentration and the land use type dominated by construction land is constantly expanding, which destroys the connectivity and integrity of the ecological landscape, resulting in an imbalance of the ecological environment and poor habitat quality. On the other hand, due to the low precipitation, high temperature and harsh natural environment in dry-hot valleys, the growth and reproduction of plants and organisms are affected, resulting in poor habitat quality. The occurrence area of native tree species forms holes in the soil and water conservation land type, destroying the continuity and integrity of the ecological barrier. Extensive economic development has destroyed the ecological environment of dry-hot valleys. In addition, natural drought disasters such as the once-in-a-century extreme drought in Yunnan Province in 2010 have reduced arable land production, reduced water area, and frequent forest fires (Huang et al., 2013). At the same time, enclosure and deforestation have also caused serious damage to the original vegetation in dry-hot valleys. This is an important reason for the high degree of habitat degradation and low habitat quality in the occurrence area of native tree species in dry-hot valleys. The areas with low habitat degradation, good habitat quality and obvious improvement in dry-hot valleys are the alpine canyon ecological barrier zone in northwestern Yunnan on the southern edge of the Qinghai-Tibet Plateau, the mountain ecological barrier zone of Ailao Mountain-Wuliang Mountain, dry-hot valleys zone of Jinsha River, and the tropical forest ecological barrier zone on the southern border. This shows that the country has played an active role in the ecological environment protection and biodiversity maintenance in the Sichuan-Yunnan ecological barrier zone through a series of ecological civilization construction projects such as the “Natural Forest Protection Project”, “Returning Farmland to Forest and Grass Project”, and “Returning Farmland to Lake Project”. However, the urban agglomeration area in the middle of dry-hot valleys and the karst areas in the northeast and southeast are both areas with strong human activities, with high habitat degradation and poor habitat quality. The ecological quality improvement in areas with poor ecological basic conditions is obviously insufficient. Therefore, on the basis of the existing ecological red line protection in Yunnan, it is necessary to strengthen the governance of the ecologically fragile areas in dry-hot valleys. This study can provide a decision-making basis for the “ecological protection compensation mechanism” of Yunnan Province. It is recommended that the high-priority vegetation restoration areas identified in the study be included in the scope of ecological compensation to encourage local governments and communities to participate in ecological restoration. Integrate policy text big data with ecological technologies such as plant germplasm resources and high-resolution remote sensing to construct a dynamic coupling model of “policy effectiveness-ecological response”.

### 4.5 Feasibility of the coupled InVEST-MaxEnt model for tree species selection

Due to changes in the natural environment, the suitable habitats of tree species in dry-hot valleys will change accordingly, making it difficult to correctly formulate long-term tree species selection plans. Our study shows that the coupled InVEST-MaxEnt model can provide a reference for the selection of ecological restoration tree species for climate adaptation, because the coupled InVEST-MaxEnt model evaluates the survival of four native tree species in the dry-hot valleys under climate change conditions and can be extended to other ecosystem type areas in Yunnan Province. However, before applying this method, the prediction results of the coupled InVEST-MaxEnt model must be verified by field experiments to determine whether these tree species can reproduce in addition to survival under future temperature and rainfall conditions. The sensitivity of *O. schwerinae*, *P. emblica*, *Q. franchetii* and *S. delavayi* to adapt to different drought climates is different, and their potential utility for survival under climate change adaptation is different. The predicted probabilities of past, future, and current suitable habitats for the four tree species are positively correlated, indicating that they can adapt well to future climate conditions. These four native tree species can continue to be used in ecological restoration areas related to dry-hot valleys in the future. Some other modeling studies have reached similar conclusions, predicting that suitable habitats for tree species will decrease or increase after transferring their SDMs to climate change scenarios. These predictions can help make decisions on protecting, managing, and restoring forest ecosystems in the face of future environmental conditions. However, to our knowledge, our study is the first attempt to validate these predictions through laboratory simulated drought experiments and field surveys to make them applicable to the early life cycle and mature stages of trees (Aguilar-Soto et al., 2015; Mair et al., 2017).

In order to verify whether this area can be used for the delineation of core planting areas, we conducted a field survey in Yuanmou County, a typical area of the hotspot area, and found that these tree species can survive and regenerate naturally in extremely difficult areas of afforestation in Yuanmou County, and the actual habitat environment is slightly different from the simulation results. However, the actual survey results are for the plant growth stage, which does not include the entire plant growth and maturity stage, which is far from enough to support the prediction of the MaxEnt model. In addition, these modeling programs can only estimate the possibility that different habitats contain the basic climatic niche of tree species-that is, the proportion of niches composed of climatic conditions that allow individuals to survive, without considering their biological interactions with other species, and cannot explain whether these habitats contain the climatic regeneration niche of tree species-that is, a series of climatic conditions that allow seeds to germinate, which is usually narrower than the climatic basic niche of the species (Soberón and Peterson, 2004; McLaughlin and Zavaleta, 2012).

In this way, assuming that the impact of natural enemies on key plant species is negligible and that the species has no dispersal restrictions, the coupled InVEST-MaxEnt model calibrated with bioclimatic variables only needs to estimate its survival probability in the entire geographic space, but the model cannot estimate its reproduction probability because this requires the calibration of the coupled InVEST-MaxEnt model with bioclimatic variables that constitute its reproductive niche (Elith et al., 2006). The differences may include that seed germination is more sensitive to soil pH, salinity and microbial community than adult plant survival; the seedling stage is susceptible to pathogen infection or competition with herbaceous plants, and the model does not incorporate such biological limiting factors. This difference between the basic niche and the actual niche requires consideration of future climate change species habitat changes and field surveys to verify the predictions of the MaxEnt model when considering the selection of suitable tree species for ecological restoration in dry-hot valleys. Under drought stress, some tree species (such as Phyllanthus emblica) have significantly stronger adaptability in the adult stage than in the germination stage through phenotypic plasticity (such as stomatal regulation and osmotic material accumulation), resulting in a phased deviation between the model prediction (based on the distribution of adult plants) and the germination experiment results. In addition, the model-predicted suitable habitat (km²scale) contains heterogeneous microhabitats, while the germination experiment (laboratory homogeneous conditions) cannot fully simulate the buffering effect of natural habitats (such as shading and litter water retention).

The actual survey results show that all four native tree species can be distributed in Yuanmou County. The core planting area of native tree species in Yuanmou County is 752.60 km^2^, which is 504.35 km^2^ more than the actual distribution area. This area may be a difficult planting area in dry-hot valleys, or a survival area for other native tree species. The prediction results of the coupled InVEST-MaxEnt model are supported by the experimental results. Under simulated drought conditions, *O. schwerinae*, *P. emblica*, *Q. franchetii* and *S. delavayi* can all germinate successfully. The probability of occupancy of the suitable area of *O. schwerinae*, *S. delavayi* and *Q. franchetii* in Yuanmou County will increase, and the probability of occupancy of the suitable area of *P. emblica* will decrease. However, it should be noted that these experimental results were only obtained at a certain point in a certain year in the geographical space. Therefore, their applicability at a larger temporal and spatial scale may be questioned, which is exactly what is needed to support the estimation of sustainable development indicators. Nevertheless, since species tend to maintain their essential and reproductive niches in space and time, i.e., changes in their survival and reproduction requirements are uncommon, it can be assumed that these experimental results apply to their entire range, both in the current climate and under future climate change scenarios (Wiens et al., 2010).

### 4.6 Regional risks and countermeasures for adaptability of native tree species in dry-hot valleys

Heavy rains, floods, droughts, extreme high temperatures, and extreme low temperatures caused by extreme weather will have an impact on dry-hot valleys ecosystem indicators (normalized vegetation index, leaf area index, etc.), plant growth distribution, number of species, structural composition, migration Ability, etc. have many impacts. In small-scale space, it is necessary to consider the impact of water and heat redistribution caused by altitude, slope and other topography on plant distribution. After comparing suitable tree species with screened out tree species, it was found that the former have stronger resistance and adaptability, and the plants are more resistant to stress. The genetic diversity of species with larger suitable areas is also significantly higher than that of some unique potential species. Suitable tree species will produce a variety of phenotypes to adapt to their environment. In the future, technologies such as genome sequencing can be applied to species distribution model predictions to improve the accuracy and innovation of the model (McLaughlin and Zavaleta, 2012). Since this study did not consider factors such as altitude, slope aspect, and extreme weather, it may cause errors in the prediction of suitable areas for plant distribution. The predicted range of potential suitable areas for plants may be wider than the actual distribution range. The potential geographical distribution of species is not limited to climate and topography. Climate complexity not only involves temperature and precipitation, but also affects light radiation intensity, soil C cycle, ozone layer changes, etc. At the same time, biological factors such as competition and reproduction, human activities, and other non-biological factors and physical barriers will also affect plant growth (Blois et al., 2013). Therefore, in future studies, we can make accurate predictions based on the physiological and biochemical effects of plants themselves, interactions between organisms, ecosystem changes, and human factors to improve the accuracy of the model. In addition, soil conditions and human management measures present a multi-dimensional dynamic interaction in the process of vegetation restoration. The physical and chemical properties of soil (such as texture, nutrient availability, and degree of salinization) constitute the material basis for vegetation reconstruction. As a key management method, the timeliness and accuracy of artificial irrigation strategies also affect soil dynamics, especially in arid ecosystems, which can effectively alleviate surface water stress. Long-term human management needs to adapt to the stage characteristics of vegetation restoration: the introduction period of pioneer species needs to focus on rapid soil improvement and water guarantee, while the near-natural succession stage should reduce human intervention and rely on soil self-repair processes driven by litter accumulation and root activity. In the future, it is necessary to break through the limitations of single measures, develop a full-chain technology system of “coupled model prediction-soil diagnosis-intelligent irrigation-vegetation regulation”, and strengthen the research on the role of microbial community functions in soil-water synergy to provide theoretical support for precise ecological restoration.

However, it must be emphasized that the optimal tree species selection proposed in this study is based on the background of ssp245 climate change. The results show that it is feasible to use the MaxEnt model to select suitable tree species in dry-hot valleys. However, the roadmap for screening suitable tree species based on the MaxEnt model is not the best choice for protecting biodiversity, controlling soil erosion and increasing forest coverage in dry-hot valleys. In the past, suitable species used to formulate ecological restoration plans were generally based on literature reviews and relevant expert opinions. The adaptability of suitable tree species may not ensure the feasibility of suitable tree species selection programs in the next few decades. A single method is difficult to apply to all areas of dry-hot valleys (Xu et al., 2023). Therefore, the prediction of the model should be combined with data such as field surveys and forest resource monitoring, and the best suitable tree species should be determined according to local conditions. Local foresters or forest management agencies should be consulted to provide strong support for the selection of suitable tree species in dry-hot valleys. It is suggested that relevant departments can adjust the afforestation plan of native tree species in dry-hot valleys area based on the MaxEnt prediction results; use the InVEST model to evaluate the ecological restoration potential of different regions and formulate zoning control measures. Combined with the research results, the national land space planning can be optimized to give priority to the restoration of high ecological value areas.

## 5 Conclusion

The optimized MaxEnt model demonstrates robust predictive capacity for mapping habitat suitability of four native tree species (*O. schwerinae, P. emblica, Q. franchetii*, and *S. delavayi*) in dry-hot valleys. Under current climatic conditions, suitable habitats exhibit fragmented distributions along mountainous corridors and riparian zones, while habitat quality hotspots predominantly cluster in central, southeastern, and northeastern valley regions. Critical planting zones (752.6 km²) overlap with intensive agricultural landscapes, necessitating strategic restoration planning to mitigate land-use conflicts. Field validation in Yuanmou County confirms species viability in predicted areas, though actual distributions marginally exceed model projections, highlighting microhabitat adaptability unaccounted for in climate-driven models.

This study advances conventional vegetation restoration paradigms by integrating InVEST-MaxEnt modeling with empirical drought tolerance assays, addressing a critical gap in climate-forward decision-making. Unlike traditional approaches reliant on expert opinion or historical records (Gastón et al., 2014), our framework explicitly quantifies species’ climate regeneration niches, providing actionable insights for multi-decadal restoration strategies. The methodology’s scalability supports its application in China’s “Dual Carbon” targets and ecological redline policy implementation, particularly in balancing afforestation goals with agricultural landscapes in Yunnan’s ecotones. It is recommended that relevant departments give priority to the highly suitable tree species identified in this study when formulating ecological restoration plans to improve the success rate of restoration, provide a decision-making basis for the ‘ecological compensation policy’, and promote sustainable ecological management in dry-hot valleys of Yunnan.

## Data Availability

The datasets presented in this study can be found in online repositories. The names of the repository/repositories and accession number(s) can be found below: www.worldclim.com.
